# Nanoparticle-induced inflammation and fibrosis in ex vivo murine precision-cut liver slices and effects of nanoparticle exposure conditions

**DOI:** 10.1007/s00204-021-02992-7

**Published:** 2021-02-08

**Authors:** Roberta Bartucci, Alex Z. van der Meer, Ykelien L. Boersma, Peter Olinga, Anna Salvati

**Affiliations:** 1 Department of Nanomedicine & Drug Targeting, Groningen Research Institute of Pharmacy, University of Groningen, A. Deusinglaan 1, 9713 AV Groningen, The Netherlands; 2 Department of Pharmaceutical Technology and Biopharmacy, Groningen Research Institute of Pharmacy, University of Groningen, A. Deusinglaan 1, 9713 AV Groningen, The Netherlands; 3 Department of Chemical and Pharmaceutical Biology, Groningen Research Institute of Pharmacy, University of Groningen, A. Deusinglaan 1, 9713 AV Groningen, The Netherlands

**Keywords:** Liver slices, Ex vivo, Corona-coated nanoparticles, Aging, Inflammation, Fibrosis

## Abstract

Chronic exposure and accumulation of persistent nanomaterials by cells have led to safety concerns on potential long-term effects induced by nanoparticles, including chronic inflammation and fibrosis. With this in mind, we used murine precision-cut liver tissue slices to test potential induction of inflammation and onset of fibrosis upon 72 h exposure to different nanomaterials (0–200 µg/ml). Tissue slices were chosen as an advanced ex vivo 3D model to better resemble the complexity of the in vivo tissue environment, with a focus on the liver where most nanomaterials accumulate. Effects on the onset of fibrosis and inflammation were investigated, with particular care in optimizing nanoparticle exposure conditions to tissue. Thus, we compared the effects induced on slices exposed to nanoparticles in the presence of excess free proteins (in situ), or after corona isolation. Slices exposed to daily-refreshed nanoparticle dispersions were used to test additional effects due to ageing of the dispersions. Exposure to amino-modified polystyrene nanoparticles in serum-free conditions led to strong inflammation, with stronger effects with daily-refreshed dispersions. Instead, no inflammation was observed when slices were exposed to the same nanoparticles in medium supplemented with serum to allow corona formation. Similarly, no clear signs of inflammation nor of onset of fibrosis were detected after exposure to silica, titania or carboxylated polystyrene in all conditions tested. Overall, these results show that liver slices can be used to test nanoparticle-induced inflammation in real tissue, and that the exposure conditions and ageing of the dispersions can strongly affect tissue responses to nanoparticles.

**Supplementary Information:**

The online version contains supplementary material available at 10.1007/s00204-021-02992-7.

## Introduction

Long-term in vivo biodistribution studies have suggested that nanomaterials may accumulate and persist at cellular level, giving rise to the question on the potential induction of chronic effects (Nel 2006; Krug 2014; Armstead and Li 2016; Devasena 2017; Landsiedel et al. 2017). Nanomaterials that are not excreted by renal or hepatobiliary elimination have been found to remain in the blood circulation and accumulate over time, mainly in the liver (Poon et al. 2019). The liver can sequester around 30–99% of nanomaterials from the systemic circulation, and long-term studies have shown that nanomaterials can be retained in this organ for months or even years (Zhang et al. 2016; Tsoi et al. 2016). Accumulation over time of nanomaterials might result in oxidative stress and DNA damage at cell level, ultimately leading to inflammation and cell death in the tissue (Rim et al. 2013; Ajdary et al. 2018). All this can cause lifelong pathological conditions, such as fibrosis (Hong and Zhang 2016; Yu et al. 2017; Lee et al. 2018). Fibrosis is characterized by the excessive production of extracellular matrix proteins by activated fibroblasts. These cells can be activated by transforming growth factor beta-1 (TGF-β1) and other inflammatory cytokines released by immune cells, including Kupffer cells, the resident liver macrophages (Zeisberg and Kalluri 2013). Indeed, nanoparticle distribution studies have shown that the mononuclear phagocyte system plays a key role in the clearance of nanomaterials, and within the liver, the Kupffer cells accumulate high amounts of nanoparticles (Ogawara et al. 1999; Sadauskas et al. 2007; De Jong et al. 2008; Dragoni et al. 2012; Gustafson et al. 2015). Additionally, some in vivo studies have already reported that some nanomaterials, such as TiO_2_ and SiO_2_, can induce fibrosis (Chen et al. 2009; Hong and Zhang 2016; Yu et al. 2017).

Within this context, in this work we aimed to test potential induction of inflammation and fibrosis in the liver upon long-term exposure to nanomaterials. In vivo models are generally considered and are still used as the gold standard to conduct chronic nanotoxicity studies (Arts et al. 2007; Park and Park 2009; Xie et al. 2010; Chan et al. 2017). However, recent efforts have been focused on the development of in vitro models (including for the liver) that could allow chronic exposure for screening of potential long-term effects induced by nanoparticles (Godoy et al. 2013; Wick et al. 2015; Usta et al. 2015; Starokozhko and Groothuis 2018). Therefore, the lifetime of cell cultures, co-cultures and 3D-organoids has been optimized to be extended up to weeks, as well as to allow the use of lower doses, to capture subtle effects induced by nanoparticles in chronic settings (Thurnherr et al. 2011; Alépée 2014; Drasler et al. 2017). However, resembling the complexity and architecture of real liver tissue remains a challenge (Materne et al. 2013; Starokozhko and Groothuis 2017, 2018). In this regard, precision-cut tissue slices are well established as a promising alternative model that allows to maintain the original architecture and complexity of real tissue (Parrish et al. 1995; Graaf et al. 2007; de Graaf et al. 2010). In particular, liver slices represent a very interesting ex vivo model for nanosafety studies, considering the key role that the liver has in relation to the fate of nanomaterials (Dragoni et al. 2012; Olinga and Schuppan 2013). Additionally, liver tissue slices can also be used as a model for the onset of fibrosis (Olinga et al. 2001; Westra et al. 2014a, 2016), since during culture they spontaneously overexpress early-fibrosis markers, e.g., heat shock protein 47 and pro-collagen 1, resembling the onset of the disease. Furthermore, when stimulated with pro-fibrotic (TGF-β1 or PDGF-β) and pro-inflammatory (LPS) factors, the diseased status appears exacerbated and stronger inflammatory responses are observed (Westra et al. 2013, 2014b).

Because of these reasons, and to test nanomaterials directly on full tissue, in this work we have selected liver slices as an advanced 3D ex vivo model to test the potential induction of inflammation and fibrosis in liver tissue upon exposure to nanomaterials. Nanoparticles were exposed to mouse liver slices for 72 h (currently set as the longest culture time with preserved tissue functions) and changes in the expression levels of a panel of markers of inflammation were monitored. The expression of fibrosis markers was also quantified, combined with collagen staining of tissue sections. Even though the exposure time was limited to 72 h, given the spontaneous onset of fibrosis in the tissue slices during culture (Westra et al. 2014b, 2016), tissue slices allowed us to test eventual differences upon exposure to nanoparticles, as a sign of nanoparticle-induced effects on the onset of fibrotic responses.

In doing so, we paid particular attention to additional effects of nanoparticle exposure conditions on the outcomes observed at tissue level. This is a key aspect to be considered, because the exposure conditions can strongly affect nanoparticle interactions with cells and tissue. When nanoparticles are tested, a representative biological fluid has to be included, since, once applied in vivo*,* proteins and other biomolecules from the environment in which nanoparticles are applied adsorb on their surface, forming the so-called protein corona. This layer completely alters the behavior of nanomaterials at cell and organism levels (Lesniak et al. 2012; Monopoli et al. 2012; Wang et al. 2013b; Duan et al. 2015). Thus, for nanoparticle exposure to liver, a source of serum proteins must be included to avoid artifacts. With this in mind, as a first approximation, in the current study nanoparticles were added to the tissue slices in medium supplemented with 5% fetal bovine serum. Additionally, to differentiate potential effects on the tissue due to the presence of serum, liver slices were exposed both to nanoparticles with the excess free proteins left in solution (in situ) or to isolated corona-coated nanoparticles in serum free conditions (Jasbi and Dorranian 2016; Lu et al. 2017). Slices exposed to nanoparticle dispersions refreshed daily were also included for comparison. It is in fact known that dispersions in biological fluids can age, and not only the corona composition may change over time (for instance due to cellular proteins excreted by cells) (Casals et al. 2010; Lundqvist et al. 2011; Lesniak et al. 2012; Hadjidemetriou and Kostarelos 2017; Giau et al. 2019), but also stability may be impaired, and agglomeration and/or settling of nanoparticles could complicate the outcomes (Cho et al. 2011; Lesniak et al. 2012; Kastl et al. 2013; Feliu et al. 2017; Böhmert et al. 2018).

Overall, using tissue slices we have monitored activation of inflammation and possible appearance of fibrosis on full liver tissue upon exposure to nanomaterials, and tested explicitly how exposure conditions and ageing of the nanoparticle dispersions in biological conditions affect the outcomes.

## Materials and methods

### Animals

Livers were extracted from C57BL/6J mice aged 6–10 weeks, both male and female. Mice had access to food and water ad libitum and were kept under a 12-h day/night cycle (Central Laboratory Animal Facility, UMCG, Groningen). Animals were allowed to acclimatize for at least 1 week before starting the experiments. Mice were sedated with 5% isoflurane in O_2_ and livers were extracted in a terminal procedure. The organ was stored in University of Wisconsin solution (UW) (DuPont Critical Care) on ice until further use. All experiments were approved by the Institutional Animal Care and Use Committee of the University of Groningen (Approval number: Dec 6416AA-001).

### Liver slice preparation

Liver slices were prepared as described earlier by De Graaf et al. (2010). Briefly, 5 mm diameter tissue cores were prepared using a 5 mm disposable biopsy puncher (Integra Miltex) and kept in ice cold UW organ preservation solution. A Krumdieck Tissue Slicer MD6000 (Alabama R&D) was filled with ice-cold Krebs–Henseleit buffer supplemented with 25 mM d-glucose (Merck), 25 mM NaHCO_3_ (Merck), 10 mM HEPES (MP Biomedicals), and saturated with a mixture of 95% oxygen and 5% CO_2_. Tissue slices were cut with a thickness of 250–350 µm, and a wet weight of roughly 5 mg. After the cutting procedure, liver slices were transferred to ice-cold UW until further use.

### Pre-incubation

Prior to experiments, liver slices were transferred to a petri dish containing William’s Medium E + GlutaMAX (WME, with l-glutamine, Invitrogen) medium supplemented with 25 mM d-glucose and 50 µg/ml gentamycin (Invitrogen) to remove the UW solution. Next, liver slices were transferred to individual wells in a 12-well plate filled with 1.3 ml pre-warmed (37 °C) serum-free WME medium or WME medium supplemented with 5% v/v Fetal Bovine Serum (FBS, Gibco from TermoFisher Scientific), saturated with 80% O_2_/5% CO_2_. Finally, the slices were maintained in an incubator (Panasonic) at 37 °C saturated with 80% O_2_/5% CO_2_ and under gentle shaking (90 rpm) for 3 h, prior to exposure to the nanoparticles. The 3 h pre-incubation allows the tissue to restore its function and decreases the presence of residual cell debris present on the edge of the slices after the cutting procedure, which could affect the subsequent exposure to nanoparticles (Bartucci et al. 2020).

### Preparation of nanoparticle dispersion and isolation of corona-coated SiO_2_ and PS-COOH nanoparticles

Far-red labelled 40 nm carboxylated polystyrene nanoparticles (FluoSpheres, PS-COOH, maximum excitation at 660 nm and emission at 680 nm) were purchased from Thermo Fisher Scientific; unlabeled 50 nm amino-modified polystyrene nanoparticles (PS-NH_2_) were purchased from Bangs Laboratories; red labelled 50 nm plain silica dioxide nanoparticles (SiO_2_, maximum excitation at 569 nm and emission at 585 nm) were purchased from Kisker Biotech; unlabeled 8 nm titanium dioxide anatase nanoparticles (TiO_2_) were purchased from PlasmaChem.

Nanoparticle dispersions were prepared during tissue slice pre-incubation and used immediately after preparation. After vortexing the nanoparticle stock for 3 min, dispersions at different concentrations were prepared by serial dilutions. Nanoparticle dispersions were prepared in serum-free WME (SF-WME) or in WME + 5% FBS (in situ). Corona-coated particles were isolated as follows, and added to tissue in serum-free medium. To prepare corona-coated PS-COOH and SiO_2_, 200 µg/ml nanoparticles were first dispersed in high serum content (40% FBS, roughly corresponding to 16 mg/ml proteins) in Dulbecco’s phosphate buffered solution (DPBS, ThermoFisher Scientific). This dispersion was incubated for at least 1 h in the dark at 37 °C while gently shaking at 250 rpm. Afterwards, the dispersion was centrifuged for 1 h at 20.000 rcf for PS-COOH or 16.000 rcf for SiO_2_ to pellet the corona-coated nanoparticles. The supernatant containing excess free proteins was discarded immediately and the pelleted corona-nanoparticle complexes were carefully resuspended in 200 µl DPBS by pipetting up and down until no pellet was visible. The dispersion was then centrifuged at 16.000 rcf for 30 s to check if the pellet was dispersed sufficiently. If pellet formation was observed, the dispersion was pipetted up and down again and centrifugation at 16.000 rcf for 30 s was repeated. If no pelleting was observed, the corona-nanoparticle complexes were considered sufficiently dispersed [as also tested by dynamic light scattering (DLS)—as described below]. Serum-free medium was added to reach the highest final nanoparticle concentration of 100 µg/ml. From here, samples at different nanoparticle concentration, down to 25 µg/ml were prepared by serial dilution. To verify that homogenous corona-nanoparticle dispersions were obtained, DLS was used to determine the size distribution of the samples obtained in each independent experiment.

### DLS measurement

The nanoparticle dispersions in relevant buffers were characterized by dynamic light scattering (DLS) using a Malvern Zetasizer Nano ZS (Malvern Instruments Ltd). Briefly, 100 μg/ml PS-COOH, PS-NH_2_, SiO_2_ and TiO_2_ nanoparticles in WME medium supplemented with 5% v/v FBS were prepared by dilution of the nanoparticle stocks and measured immediately after dispersion. In addition, 100 μg/ml corona-coated nanoparticle dispersions in serum-free WME prepared as described above were also characterized by DLS. The results are the average of three separate measurements, each containing 10 runs of 10 s.

### Exposure to nanoparticles

After a 3 h pre-incubation, liver slices were exposed to nanoparticles by transferring them to pre-warmed (37 °C) and pre-saturated (80% O_2_/5% CO_2_) wells containing nanoparticle dispersions at different doses in serum-free WME medium or WME medium supplemented with 5% v/v FBS, prepared as described above. Then, the liver slices were maintained in culture up to 72 h exposure in the same medium. To compare results in slices exposed to daily refreshed medium, for some samples the medium and/or the freshly prepared nanoparticle dispersion or corona-isolated nanoparticles were replaced every 24 h.

### Viability of liver slices

The viability of liver slices was determined based on adenosine triphosphate (ATP) content normalized by total protein content. The ATP content of the slices was determined using an ATP bioluminescence assay (Sigma Aldrich). The amount of light produced correlates directly to the amount of ATP present, which allows calculation of ATP content using a calibration curve. After exposure, slices were snap-frozen in sonification solution (SONOP) with pH 10.9. SONOP is constituted by 2 mM EDTA and 70% v/v ethanol. Then, the slices were thawed on ice and homogenized for 45 s using a mini-beadbeater-24 (Biospec). An ATP calibration curve was prepared from ATP standards and 50 µl of each sample at different concentration were transferred in duplicate in a black 96-well plate (Costar). Next, the homogenized samples were centrifuged at 16,100 rcf for 5 min at 4 °C. Part of the supernatant was transferred to new safe-lock Eppendorf cups and the cups with the pellet were dried overnight at 37 °C for determination of the protein content. Next, 5 µl of the transferred supernatant was pipetted in duplicate in a 96-well plate and diluted with 45 µl 100 mM Tris–HCl (pH 7.6–8.0) (VWR International Prolabo), with 2 mM EDTA. Finally, 50 µl of the luciferase/luciferin mix was added to each well and luminescence (relative fluorescence units, RFU) was measured immediately and after 5 min using a luminescence microplate reader (LumiCount BL10000, Packard). Calculations were performed using the RFU at 5 min.

The total protein content of the samples was determined using a colorimetric protein assay (DC protein assay, Bio-Rad). Two hundred μl of 5 M  NaOH was added to the dried pellets in Eppendorf cups from the ATP assay and incubated for 30 min in a water bath at 37 °C while gently shaking at 100 rpm. Meanwhile, a protein calibration curve was made from bovine serum albumin (BSA, ICN Biomedicals Inc.) in 1M NaOH. After incubation for 30 min, the samples were diluted to 1M NaOH by adding 800 µl ultrapure water and then homogenized for 45 s using a mini-beadbeater. Five µl of each standard dilution for the calibration curve and of each sample were pipetted in duplicate in a clear, flat bottom 96-well plate. A multichannel pipet was used to add 25 µl of reagent A and 200 µl reagent B to all filled wells. The plate was stored in the dark for 15 min and finally the absorbance at 650 nm was measured using a microplate reader (THERMOmax microplate reader, Molecular Devices).

Finally, for each slice the viability was calculated by normalizing the ATP value (pmol) by the amount of total protein (μg). For each condition three slices were used, and the average and standard error of the mean were calculated. Supplementary Figures S2, S5, S8 and S12 show the results of the three independent experiments, together with their mean (indicated with a line) and standard error of the mean.

### mRNA extraction, cDNA synthesis and qRT-PCR

For mRNA extraction, for each sample three slices were pooled together. Samples for mRNA expression analysis were transferred to 1.5 ml cups containing glass beads and a homogenization solution supplemented with 1-thioglycerol from a Maxwell 16 LEV simply RNA purification kit (Promega). Next, the samples were homogenized using a mini-beadbeater and homogenates were heated to 70 °C for 2 min. A Maxwell 16 Instrument set to the simplyRNA protocol was used to isolate RNA from the processed samples into nuclease-free water. Afterwards, the RNA concentration of each sample was determined using a Nanodrop ND-1000 spectrophotometer (Thermo Fisher Scientific, the Netherlands). Subsequently, the volume of RNA solution needed to obtain 1.6 µg of isolated RNA was calculated based on the RNA concentration and nuclease-free water was added to obtain a total volume of 10 µl. If 1.6 µg of RNA could not be obtained with a volume of < 10 µl RNA solution, 10 µl of RNA was used (without further dilution) and the difference in total amount of RNA was adjusted for after cDNA synthesis. After preparation of the RNA, a cDNA synthesis reaction mix was made using an M-MLV reverse transcriptase kit (Promega). A volume of 8.5 µl reaction mix was added to each sample to a total volume of 18.5 µl, then samples were vortexed and centrifuged for 1 min to remove any bubbles. Next, the samples were placed in an Eppendorf Mastercycler Gradient Thermal Cycler and heated as follows to reverse transcribe the RNA to cDNA: 20 °C for 10 min, 42 °C for 30 min, 20 °C for 12 min, 99 °C for 5 min and 20 °C for 5 min.

An amount of 1.6 µg cDNA in 18.5 µl water was synthesized, which was then diluted to 100 ng/µl in nuclease-free water. For each gene, the cDNA of each sample was pipetted in triplicate into a 384-Well Reaction Plate (MicroAmp, Applied Biosystems). Real-time quantitative polymerase chain reaction (RT-qPCR) was used to quantify the mRNA expression using the primers described in Table 1 and a SYBR green Low-ROX Kit (SensiMix, Bioline Reagents Limited). The primers were tested for efficiency and all had an efficiency between 90 and 105%. A Quantstudio 7 flex Real-Time PCR System (Thermo Fisher Scientific) was used to run RT-qPCR with a hold stage of 10 min at 95 °C, a PCR stage of 40 cycles of 15 s at 95 °C (1.6 °C/s) and 25 s at 65 °C (1.6 °C/s). A continuous melt curve stage was included with 15 s at 95 °C (1.6 °C/s), 1 min at 65 °C (1.6 °C/s), and another 15 s at 95 °C (0.05 °C/s, dissociation phase). Data was extracted using Quantstudio Real-Time PCR software (version 1.3). Finally, the mRNA expression was calculated with the 2^−ΔΔ*Ct*^ method, with β-actin used as a reference gene, to obtain relative fold induction values as follows:

$$\Delta Ct \, = \, Ct_{{{\text{gene}}}} - Ct_{{{{\beta{\text{-actin}}}}}}$$

$$\Delta \Delta Ct \, = \, \Delta Ct_{{{\text{treated}}}} - \Delta Ct_{{{\text{untreated}}}}$$

$${\text{Fold induction }} = \, 2^{ - \Delta \Delta Ct}$$

**Table 1:** Primers used for qRT-PCR

Gene coding for	Name		Sequence
β-Actin	*b-Actin*	*Fw*	5′-ATCGTGCGTGACATCAAAGA
		*Rv*	5′-ATGCCACAGGATTCCATACC
Procollagen type 1α1	*pCOL1a1*	*Fw*	5′-TGACTGGAAGAGCGGAGAGT
		*Rv*	5′-ATCCATCGGTCATGCTCTCT
Collagen type 1α1	*COL1a1*	*Fw*	5′-TGACTGGAAGAGCGGAGAGT
		*Rv*	5′-ATCCATCGGTCATGCTCTCT
Heat shock protein 47	*HSP-47*	*Fw*	5′-AGGTCACCAAGGATGTGGAG
		*Rv*	5′-CAGCTTCTCCTTCTCGTCGT
α Smooth muscle actin	*a-SMA*	*Fw*	5′-ACTACTGCCGAGCGTGAGAT
		*Rv*	5′-CCAATGAAAGATGGCTGGAA
Interleukin 1β	*IL-1b*	*Fw*	5′-GCACTACAGGCTCCGAGATGAAC
		*Rv*	5′-TTGTCGTTGCTTGGTTCTCCTTGT
Interleukin 4	*IL-4*	*Fw*	5′-GTCTGCATCAAGACGCCATG
		*Rv*	5′-CGTTGCTGTGAGGACGTTTG
Interleukin 6	*IL-6*	*Fw*	5′-TGATGCTGGTGACAACCACGGC
		*Rv*	5′-TAAGCCTCCGACTTGTGAAGTGGTA
Interleukin 10	*IL-10*	*Fw*	5′-CCCAAGTAACCCTTAAAGTCCTGC
		*Rv*	5′-ATAACTGCACCCACTTCCCAGTC

### Paraffin sections of liver slices

For paraffin embedding, slices were fixed in 4% formaldehyde in PBS for 24 h at 4 °C and stored in 70% ethanol at 4 °C until analysis. After dehydration in alcohol and xylene, the slices were embedded in paraffin and 4 μm sections were cut perpendicular to the surface of the slice using a Leica Reichert-Jung 2040 Autocut Microtome. The sections were deparaffinated and incubated overnight in a 0.1 M Tris–HCl buffer (pH 9.0 room temperature) at 80 °C for antigen retrieval.

### Collagen 1 immunostaining

After antigen retrieval, the sections were washed in 1 × PBS (phosphate buffered saline, pH 7) and circled using a DAKO pen (ImmEdge, Vector Laboratories inc.). A droplet of a 1:400 dilution of goat anti-type I collagen antibody (IgG, unlabeled, Southern Biotech) was added to the sections and incubated for 1 h at room temperature. Next, endogenous peroxidases were inhibited by incubating the sections in 0.1% H_2_O_2_ in methanol for 20 min. The sections were washed in 1 × PBS, then incubated for 30 min with a 1:100 dilution of secondary antibody (rabbit-anti-goat IgG (H + L), Mouse/Rat/Human ads-horse radish peroxidase (HRP) (Southern Biotech) in 1 × PBS with 5% normal mouse serum (NMS), and washed again. After a 30-min incubation with a 1:100 dilution of tertiary antibody [goat-anti-rabbit IgG, IgG(H + L)-HRP, Southern Biotech] in 1 × PBS with 5% NMS, the sections were washed and incubated for 5 min with a Novared reaction mixture (ImmPACT, NovaRED peroxidase substrate kit for laboratory use, Vector Laboratories). The sections were incubated in hematoxylin for 1 min, flushed with tap water for 5 min, and dehydrated in three washes with 100% ethanol. Finally, the dehydrated sections were mounted using DePex (SERVA Electrophoresis GmbH). Pictures of the stained sections were taken using an Olympus BX41 Microscope and Hamamatsu NanoZoomer digital slide scanner.

### Picro-Sirius Red staining

The presence of both collagen type I and III fibers was shown using a histochemical Picro-Sirius Red staining. Similar to the collagen type I staining, slices in paraffin blocks were cut in 4 μm sections and placed on glass slides (StarFrost, Waldemar Knittel glass). The sections were deparaffinated, incubated in hematoxylin for 30 s, flushed with tap water for 5 min, and then incubated for 1 h in an aqueous, saturated picric acid solution with 0.1 mg/ml Sirius Red (Sirius red, SCHMIDT GMBH & CO.). Afterwards, the sections were washed in two changes of acid water; 0.5% acetic acid (glacial, 100%, anhydrous, Sigma Aldrich) in ultrapure water and dehydrated in three changes of 100% ethanol. Finally, the sections were mounted using DePex and pictures were taken using Olympus BX41 Microscope.

### Statistics

All experiments were carried out in biological triplicate or more (≥ 3 animals) and each experiment included three replicate slices per condition. The results of each independent experiment are shown, together with an horizontal line for their mean and vertical lines for the standard error of the mean (SEM). The qRT-PCR results are shown as fold induction of 2^−ΔΔ*Ct*^ over the control, but Kruskal–Wallis statistic followed by Dunnett’s multiple comparisons test were performed on Δ*Ct* values using GraphPad 5.0.

## Results

### Response of the liver slices to different culture conditions

Murine liver slices were prepared as previously described and exposed to a panel of nanomaterials for up to 72 h (de Graaf et al. 2010; Westra et al. 2014b). Precision-cut liver slices cultured for more than 48 h have been shown to spontaneously develop fibrosis (Westra et al. 2014b; Pham et al. 2015). Standard markers used to follow fibrogenesis and the development of fibrosis are upregulated at gene level over time. These include for instance heat shock protein-47 (*Hsp47*), which ensures proper folding of collagen, procollagen1, precursor peptide of collagen 1, as well as collagen 1, one of the main components of the extra cellular matrix (ECM). Additionally, it has been shown that alpha smooth muscle actin (*α*-*Sma)*, used as a marker for activated fibrogenic cells, is first downregulated (likely as a response to the initial tissue damage with the sectioning of the tissue) and then increases over time during culture, suggesting activation of hepatic stellate cells (Westra et al. 2014b).

**Fig. 1:**
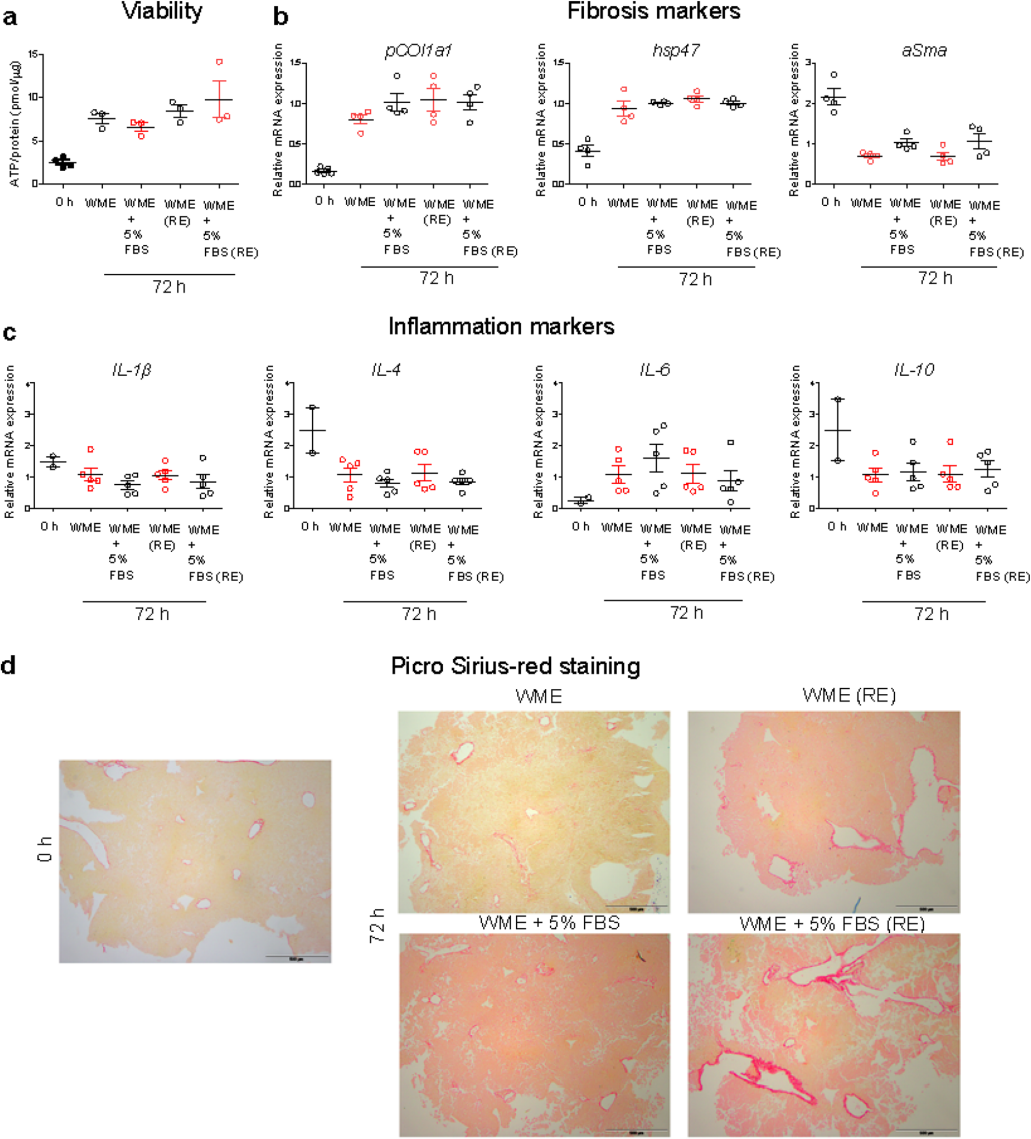
Response of tissue slices to different growth conditions. Liver slices were cultured for 72 h in serum-free medium (WME), medium supplemented with 5% FBS (WME + 5% FBS), daily-refreshed serum-free medium (WME RE) or daily-refreshed 5% FBS medium [WME + 5% FBS (RE)] and tested for viability (**a**), the expression of fibrosis and inflammatory markers (**b** and **c**, respectively) and collagen staining (**d**). **a** Viability is expressed as the ATP content (pmol) normalized by amount of total protein (µg). The results are the mean and standard error of the mean of 3–5 independent experiments. **b** and **c** The expression of selected markers is shown as fold induction over the expression levels in slices cultured in serum-free medium (without refreshing of the medium). The results are the mean and standard error of the mean of 4–5 independent experiments. The relative gene expression was determined by qRT-PCR and calculated using *β-actin* as housekeeping gene as described in the “Materials and methods” section. Kruskal–Wallis statistic followed by Dunnett’s multiple comparisons test were performed on Δ*Ct* values. **d** Paraffin sections of liver slices freshly cut (0 h), and maintained in culture for 72 h in serum-free medium (WME) or in medium supplemented with 5% FBS (WME + 5% FBS) non refreshed or daily-refreshed (RE). Red: collagen fibers. Scale bar = 500 µm

Thus, as a first step, in order to use liver tissue slices to test potential impact on inflammation and the onset of fibrosis induced by nanoparticles, the effects of different culturing conditions on the tissue were compared. Because of the need of including serum proteins when testing nanomaterials to allow corona formation (Monopoli et al. 2012; Wang et al. 2013b), tissue slices maintained in medium supplemented with 5% fetal bovine serum (FBS) were tested, together with standard serum-free conditions, usually applied for optimal tissue maintenance (Olinga et al. 2001; de Graaf et al. 2010; Westra et al. 2014b; Gore et al. 2019). Furthermore, the results obtained in slices cultured for 72 h were compared to the results in slices maintained for the same time, but with the medium refreshed daily. Tissue viability, collagen staining and the expression of a panel of classic markers of fibrosis and inflammation were determined and compared for the different conditions (Fig. 1). IL-1β and IL-6 were chosen as commonly classified pro-inflammatory cytokines, while IL-4 and IL-10 represented anti-inflammatory cytokines (Cavaillon 2001).

The results showed that the viability of liver slices increased up to 72 h compared to freshly cut slices (0 h), and no differences were observed among slices maintained in serum-free medium and medium supplemented with 5% FBS, both with and without refreshing of the medium (Fig. 1a). As expected, liver slices overexpressed the fibrosis markers *Hsp47* and *Pcol1a* over time in all cases (Fig. 1b), while the expression of α-*Sma* decreased as previously observed. No differences in gene expression of fibrosis markers were observed among slices cultured in the different conditions tested (in the presence of serum and/or with medium daily refreshed). Additionally, paraffin sections of liver slices were stained for collagen fibers using picro-sirius red staining (Fig. 1d). After 72 h of incubation, a mild increase in collagen staining was observed (Westra et al. 2014b). However, quantification of the Pro-Collagen I α1 in the medium is required to fully confirm potential activation of fibrosis (Gore et al. 2019).

Next, the expression of the inflammation markers *IL-4* and *IL-10* decreased, while the expression of *IL-6* increased, when compared to slices at *t* = 0 h (Fig. 1c).

Taken together, these results suggest that the presence of 5% FBS did not significantly alter the viability of the slices and had only minor effects on the gene expression levels of *α-Sma* and *IL-6*. No differences were detected when the culture medium was refreshed daily, nor on slices cultured in the presence of 5% FBS, possibly because this is still a relatively low serum concentration. Given that nanoparticles will accumulate in the liver via the systemic circulation in full plasma, it will be important to repeat similar studies using much higher serum concentrations. In relation to this, the use of corona-coated nanoparticles in serum-free conditions, as we tested here, may be a good strategy to use a realistic corona formed in full serum or full plasma, while at the same time avoiding additional effects on tissue responses due to the presence of such high protein content.

### Exposure to amino-modified polystyrene PS-NH_2_ nanoparticles

As a first step, liver slices were exposed to 50 nm amino-modified polystyrene (PS-NH_2_), a well-studied model nanoparticle in nanosafety for toxicity induced by some positively charged objects (Kim et al. 2012; Wang et al. 2013a). PS-NH_2_ nanoparticles are known to induce inflammation and to have different impact on cells. When exposed to cells in the presence of biological fluids, thus covered by a corona, these nanoparticles have been shown to induce apoptosis (Xia et al. 2008; Lunov et al. 2011; Wang et al. 2013a). However, they can induce fast necrosis if added to cells in artificial serum-free conditions, due to direct interactions of their positively-charged surface with the cell membrane (Wang et al. 2013a). Thus, we exposed liver slices to these nanoparticles in serum-free conditions as a control, to confirm that inflammatory responses in the tissue could be detected.

Prior to exposure to tissue, dynamic light scattering (DLS) was used to characterize the PS-NH_2_ dispersions formed in serum-free medium, as well as in medium with 5% FBS (in situ), and after isolation of corona-coated nanoparticles. The size distribution showed a mild sign of agglomeration for the dispersion in situ, but multiple peaks were observed for corona-coated nanoparticles in serum-free medium, suggesting that centrifugation for corona isolation led to particle agglomeration (Supplementary Figure S1). To avoid confusing results due to exposure to unstable dispersions or agglomerates, in this case we decided to expose liver slices only to PS-NH_2_ nanoparticles in medium supplemented with 5% FBS or—for comparison as a control—in serum-free medium, with and without refreshing the nanoparticle dispersion daily.

**Fig. 2:**
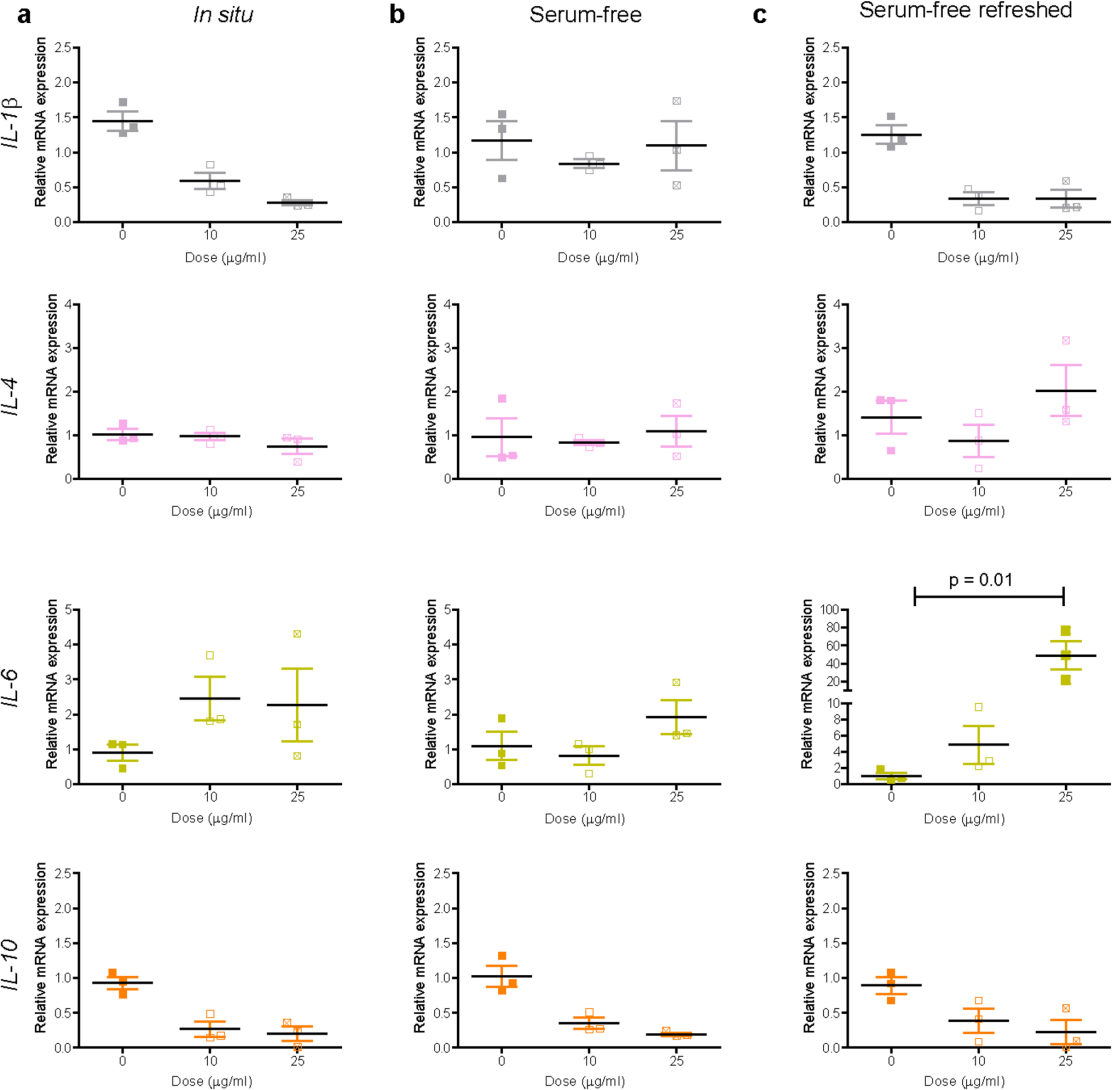
Expression of inflammation markers after PS-NH_2_ nanoparticle exposure. Liver slices were exposed for 72 h to PS-NH_2_ in WME + 5% FBS (**a**), serum-free medium (**b**) and daily-refreshed serum-free medium (**c**). The relative gene expression was determined by qRT-PCR and calculated using *β-actin* as housekeeping gene. The data are presented as the fold induction over the expression levels in slices cultured in serum-free medium (without refreshing of the medium), calculated as described in the “Materials and methods” section. The results are the mean and standard error of the mean of three independent experiments. Kruskal–Wallis statistic followed by Dunnett’s multiple comparisons test were performed on Δ*Ct* values

We previously determined loss of viability over 72 h in liver slices exposed to PS-NH_2_ nanoparticles in situ and in serum-free medium when dispersions were not refreshed (Bartucci et al. 2020). Here, the results confirmed a strong decrease in viability also in slices exposed to these nanoparticles in daily-refreshed serum-free medium (Supplementary Figure S2). In relation to inflammation markers, exposure to PS-NH_2_ nanoparticles caused downregulation of *IL-1β* and *IL-10* in all exposure conditions (Fig. 2), although the effect was not significant. Interestingly, upregulation of *IL-6* was observed in slices exposed to the nanoparticles in medium supplemented with 5% FBS, as well as in serum-free medium. However, *IL-6* expression increased up to 50-fold when slices were exposed to these nanoparticles in serum-free medium and the dispersion was refreshed daily. This confirmed that, as expected, exposure to the bare nanoparticles in serum-free conditions led to a strong inflammatory response, and additionally clear effects due to ageing of the nanoparticle dispersions were present, thus inflammation was much stronger in slices exposed to daily-refreshed nanoparticle dispersions.

**Fig. 3:**
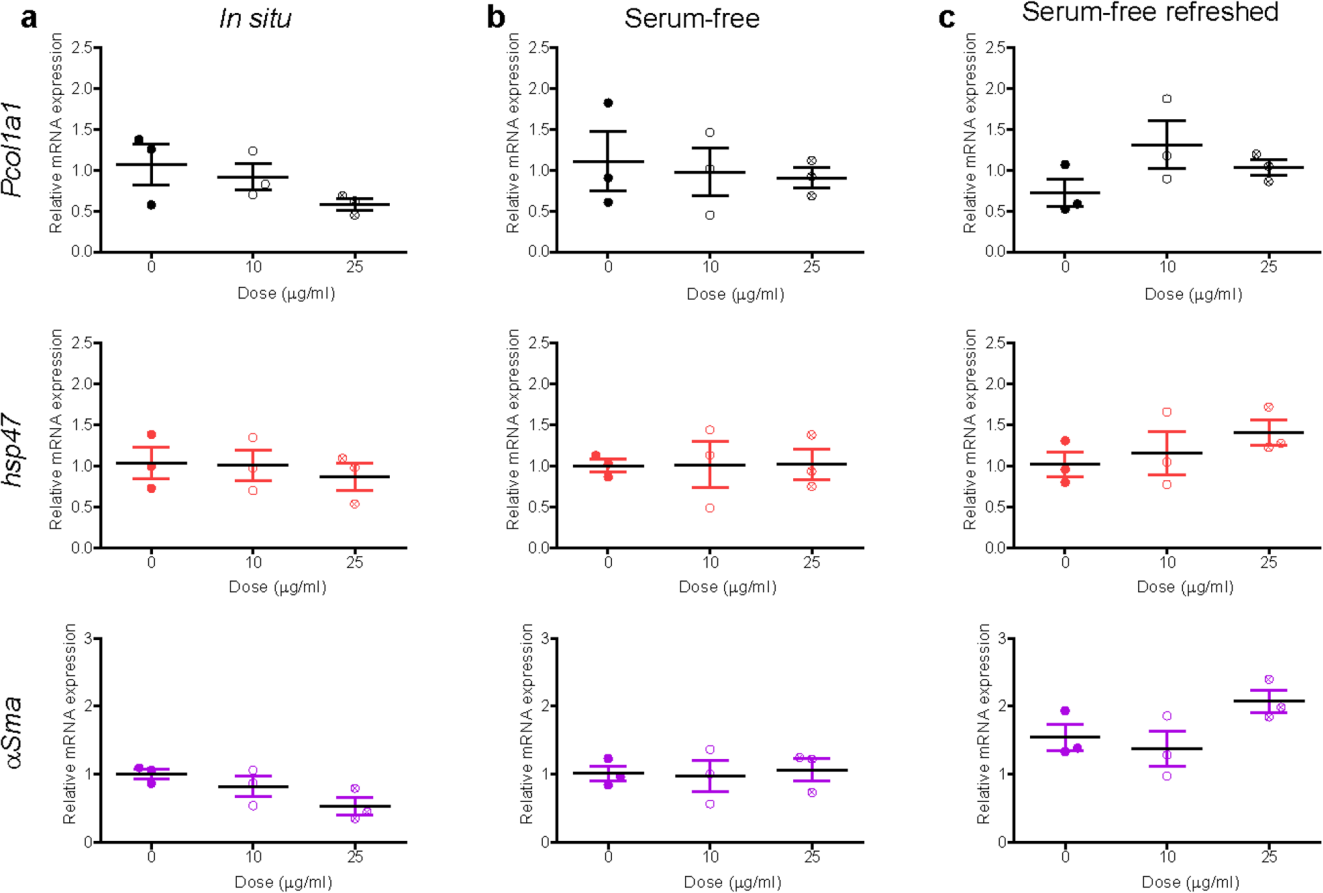
Expression of fibrosis markers after PS-NH_2_ nanoparticle exposure. Liver slices were exposed for 72 h to PS-NH_2_ in WME + 5% FBS (**a**), in serum-free medium (**b**) and daily-refreshed serum-free medium (**c**). The relative gene expression was determined by qRT-PCR and calculated using *β-actin* as housekeeping gene. The data are presented as the fold induction over the expression levels in slices cultured in serum-free medium (without refreshing of the medium), calculated as described in the “Materials and methods” section. The results are the mean and standard error of the mean of three independent experiments. Kruskal–Wallis statistic followed by Dunnett’s multiple comparisons test were performed on Δ*Ct* values.

When testing the expression of fibrosis markers, a concentration-dependent decrease in the expression of *Pcol1a1* and *αSma* was observed in slices exposed to nanoparticles in medium supplemented with 5% FBS (Fig. 3). A slight increase was observed for the expression of *Hsp47* and *αSma* with PS-NH_2_ in daily-refreshed serum-free medium, however this was not significant. Immunostaining of collagen I and sirius-red staining were also performed, and no evident alterations could be detected (Supplementary Figure S3).

Overall, these results allowed us to confirm—first of all—that tissue slices are a good model to test inflammatory responses induced by nanoparticles and—secondly—that the exposure conditions can strongly affect the response of the tissue to the nanoparticles. The presence of a corona is required to ensure realistic exposure and avoid inflammatory responses as a consequence of artificial exposure to serum-free bare nanoparticles. At the same time, additional effects due to ageing of the nanoparticle dispersion also need to be considered. Even though slices are cultured under gentle shaking (see “Materials and methods” section for details) which should limit effects due to nanoparticle sedimentation and settling of agglomerates, it is interesting to see that refreshing the nanoparticle dispersions led to much stronger effects on the slices. Similar ageing effects can have profound impact on the response of the tissue, as we observed here, and are particularly important when testing nanoparticles over a longer period.

### Exposure to PS-COOH nanoparticles

**Fig. 4:**
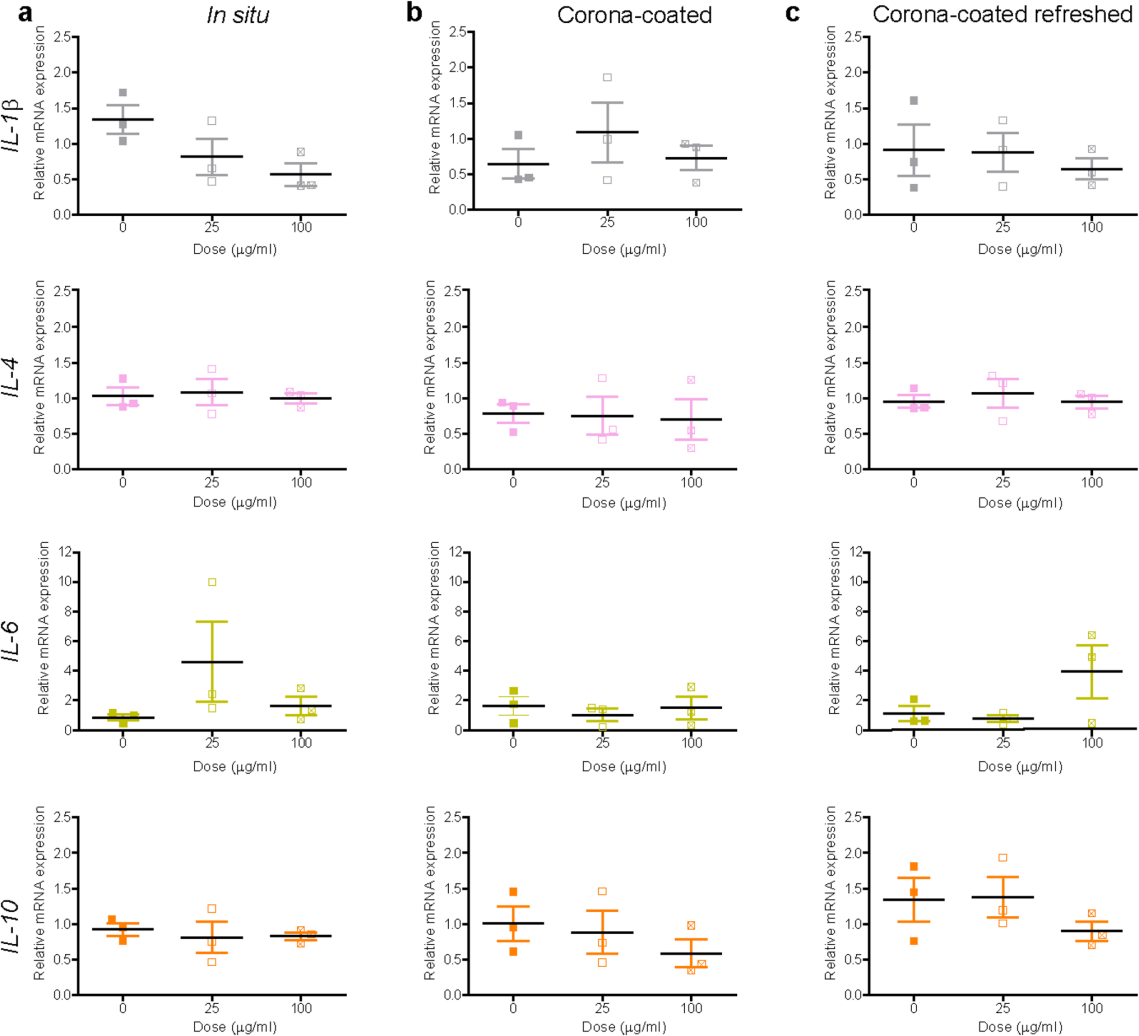
Expression of inflammation markers after PS-COOH nanoparticle exposure. Liver slices were exposed for 72 h to PS-COOH in WME + 5% FBS (**a**), and to isolated corona-coated PS-COOH in serum-free medium without (**b**) and with daily refreshment of the corona-coated PS-COOH dispersion (**c**). The relative gene expression was determined by qRT-PCR and calculated using *β-actin* as housekeeping gene. The data are presented as the fold induction over the expression levels in slices not exposed to nanoparticles, calculated as described in the “Materials and methods” section. The results are the mean and standard error of the mean of three independent experiments. Kruskal–Wallis statistic followed by Dunnett’s multiple comparisons test were performed on Δ*Ct* values

**Fig. 5:**
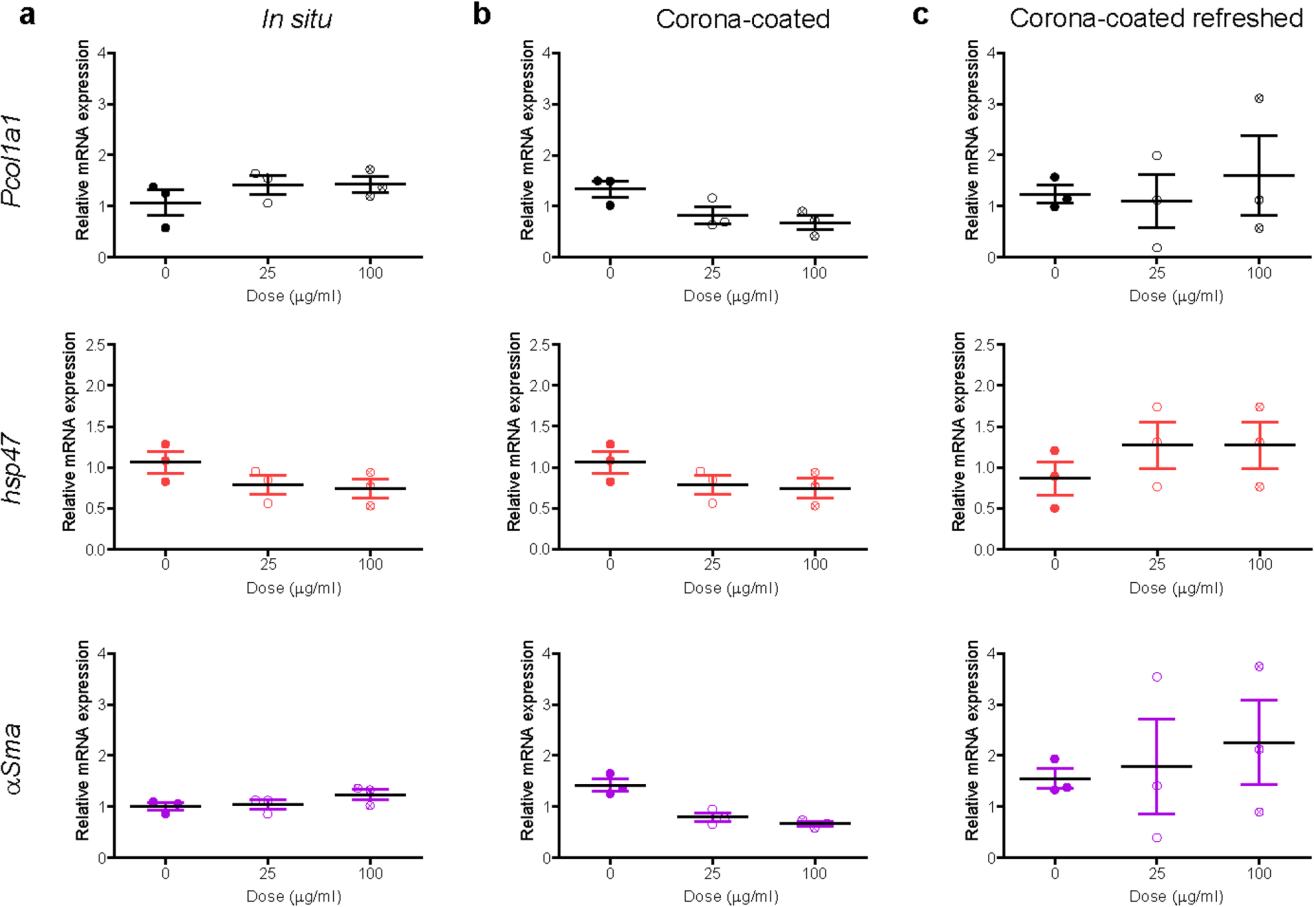
Expression of fibrosis markers after PS-COOH nanoparticle exposure. Liver slices were exposed for 72 h to PS-COOH in WME + 5% FBS (**a**), and to isolated corona-coated PS-COOH in serum-free medium without (**b**) and with daily refreshment of the corona-coated PS-COOH dispersion (**c**). The relative gene expression was determined by qRT-PCR and calculated using *β-actin* as housekeeping gene. The data are presented as the fold induction over the expression levels in slices not exposed to nanoparticles, calculated as described in the “Materials and methods” section. The results are the mean and standard error of the mean of three independent experiments. Kruskal–Wallis statistic followed by Dunnett’s multiple comparisons test were performed on Δ*Ct* values

Next, liver slices were exposed to 40 nm carboxylate-modified polystyrene (PS-COOH) nanoparticles. We previously showed by confocal microscopy and flow cytometry on cells recovered after tissue digestion that these nanoparticles are efficiently internalized in liver tissue slices, with a preferential accumulation in the Kupffer cells (Bartucci et al. 2020). PS-COOH are commonly used together with the PS-NH_2_ nanoparticles as model nanoparticles, usually considered nontoxic and toxic, respectively (Bexiga et al. 2011; Lunov et al. 2011; Loos et al. 2014). DLS data showed that homogenous dispersions could be obtained for nanoparticles dispersed in medium with 5% FBS and also after isolation of corona-coated PS-COOH (Supplementary Figure S4). Therefore, both exposure conditions were tested on tissue slices, together with daily refreshed corona-coated PS-COOH dispersions. Tissue viability appeared unaffected by exposure to the nanoparticles in all conditions (Supplementary Figure S5). In relation to inflammation, a concentration-dependent downregulation was observed for *IL-1β* when slices were exposed to PS-COOH nanoparticles in situ. An increase in the expression of *IL-6* was instead observed in slices exposed to the PS-COOH in medium with 5% FBS*,* as well as to corona-PS-COOH with the medium daily refreshed (Fig. 4). Fibrosis markers did not show any significant alteration (Fig. 5), in line with similar results by collagen staining (Supplementary Figure S6).

### Exposure to SiO_2_ nanoparticles

Next to polystyrene, amorphous 50 nm silica (SiO_2_) nanoparticles were chosen as an additional nanomaterial for this study because they are relevant for nanosafety studies and highly studied in vitro as well as in vivo for possible chronic effects (Arts et al. 2007; Park and Park 2009; Xie et al. 2010; Lesniak et al. 2012; Chan et al. 2017). We previously determined uptake of SiO_2_ nanoparticles by liver tissue slices by confocal imaging (Bartucci et al. 2020). DLS analysis showed that these nanoparticles were well monodispersed in medium with 5% FBS (Supplementary Figure S7) and, similarly, isolation of corona-coated SiO_2_ in serum-free conditions allowed us to obtain homogenous dispersions. Given the good stability of the corona-coated SiO_2_ and of the SiO_2_ nanoparticle dispersion in situ, liver slices were exposed to the nanoparticles in both conditions up to 72 h. Furthermore, the isolated corona-coated SiO_2_ nanoparticles were also tested with daily refreshment of the dispersion.

**Fig. 6:**
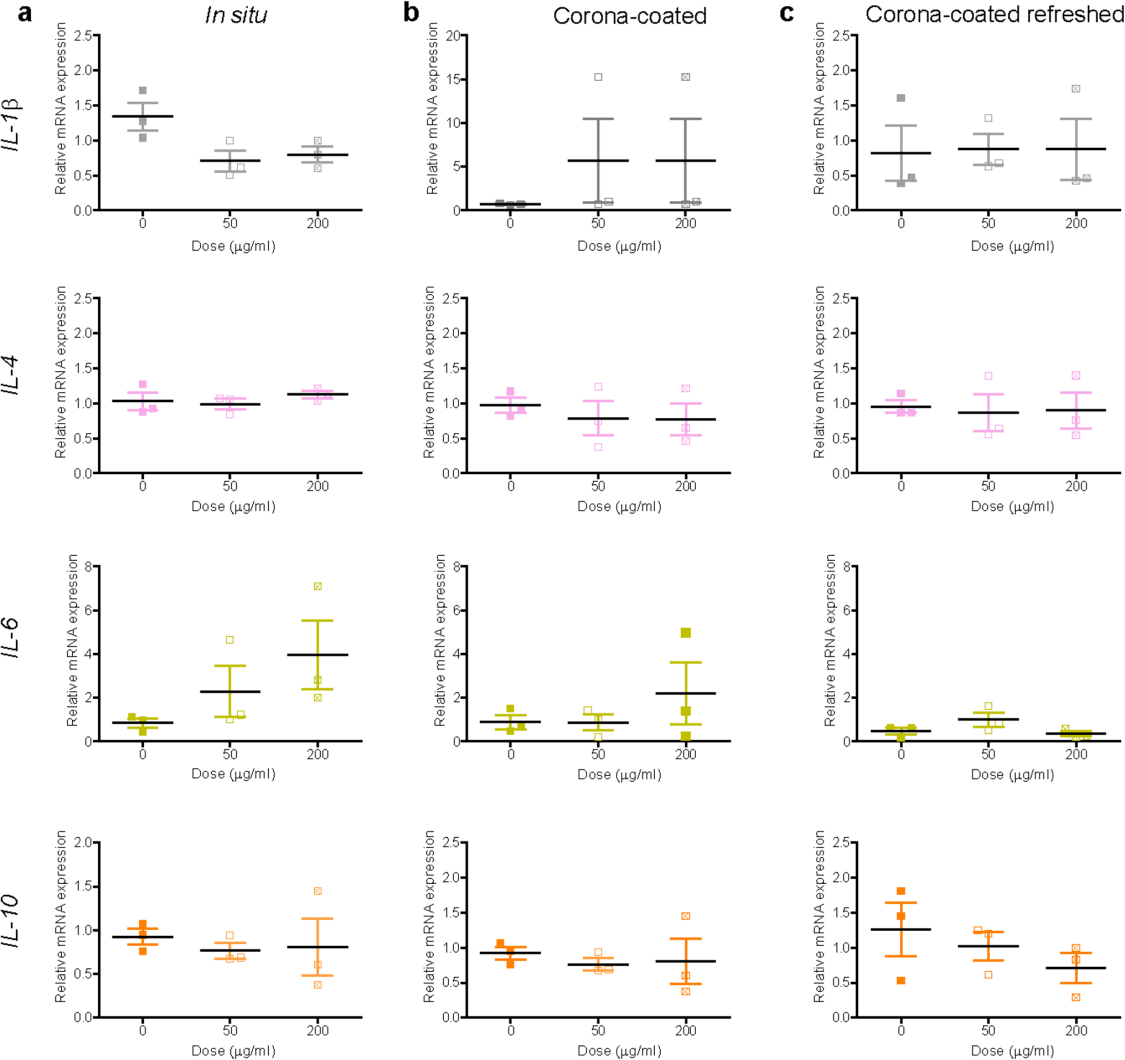
Expression of inflammation markers after SiO_2_ nanoparticle exposure. Liver slices were exposed for 72 h to SiO_2_ in WME + 5% FBS (**a**), and to isolated corona-coated SiO_2_ in serum-free medium without (**b**) and with (**c**) daily refreshment of the corona-coated SiO_2_ dispersion. The relative gene expression was determined by qRT-PCR and calculated using *β-actin* as housekeeping gene. The data are presented as the fold induction over the expression levels in slices not exposed to nanoparticle, calculated as described in the “Materials and methods” section. The results are the mean and standard error of the mean of three independent experiments. Kruskal–Wallis statistic followed by Dunnett’s multiple comparisons test were performed on Δ*Ct* values

**Fig. 7:**
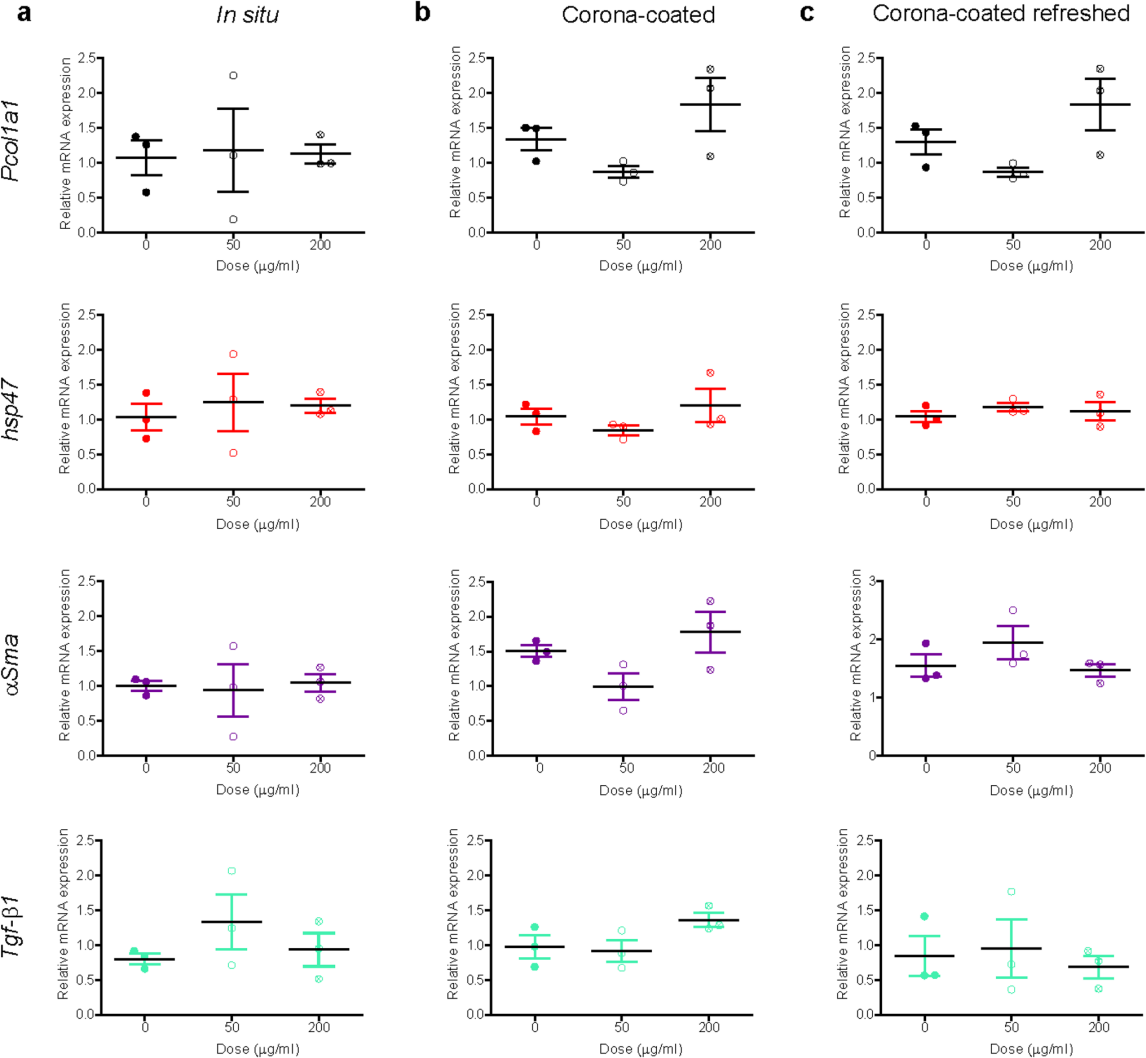
Expression of fibrosis markers after SiO_2_ nanoparticle exposure. Liver slices were exposed for 72 h to SiO_2_ in WME + 5% FBS (**a**), and to isolated corona-coated SiO_2_ in serum-free medium without (**b**) and with (**c**) daily refreshment of the corona-coated SiO_2_ dispersion. The relative gene expression was determined by qRT-PCR and calculated using *β-actin* as housekeeping gene. The data are presented as the fold induction over the expression levels in slices not exposed to nanoparticles, calculated as described in the “Materials and methods” section. The results are mean and standard error of the mean of three independent experiments. Kruskal–Wallis statistic followed by Dunnett’s multiple comparisons test were performed on Δ*Ct* values

A mild decay of viability was observed only in liver slices exposed to corona-coated SiO_2_ at the highest concentration (Supplementary Figure S8). When testing potential inflammatory responses (Fig. 6), an increase in *IL-6* expression and small decrease of *IL-1β* were observed in liver slices exposed to SiO_2_ nanoparticles in situ. A mild decrease in *IL-10* was observed in slices exposed to the highest concentration of corona-coated SiO_2_ when the dispersion was refreshed daily, while *IL-4* did not show any alteration. Similarly, analysis of fibrosis markers showed only a small increase in *Pcol1a1* expression in slices exposed to corona-coated SiO_2_ with and without refreshing of the dispersion, and a small increase in *α-Sma* expression in slices exposed without refreshment of the dispersion (Fig. 7). Next*, Tgf-β* levels were also determined and staining for collagen I was performed (Supplementary Figures S9 and S10). No significant differences were observed among all conditions tested in respect to untreated slices.

Overall, these results indicated that no significant alterations in the tested inflammation and fibrosis markers were detected in liver slices exposed to silica nanoparticles. However a previous in vivo study showed that amorphous SiO_2_ nanoparticles can lead to fibrosis in the liver via oxidative stress, which in turn causes persistent inflammation and TGF-β1/Smad3 activation (Yu et al. 2017). This in vivo study was performed over a period of 15–60 days. The much longer time-scale may suggest that in our case the exposure time was too short to observe similar changes at mRNA and protein levels in the tissue.

### Exposure to TiO_2_ nanoparticles

**Fig. 8:**
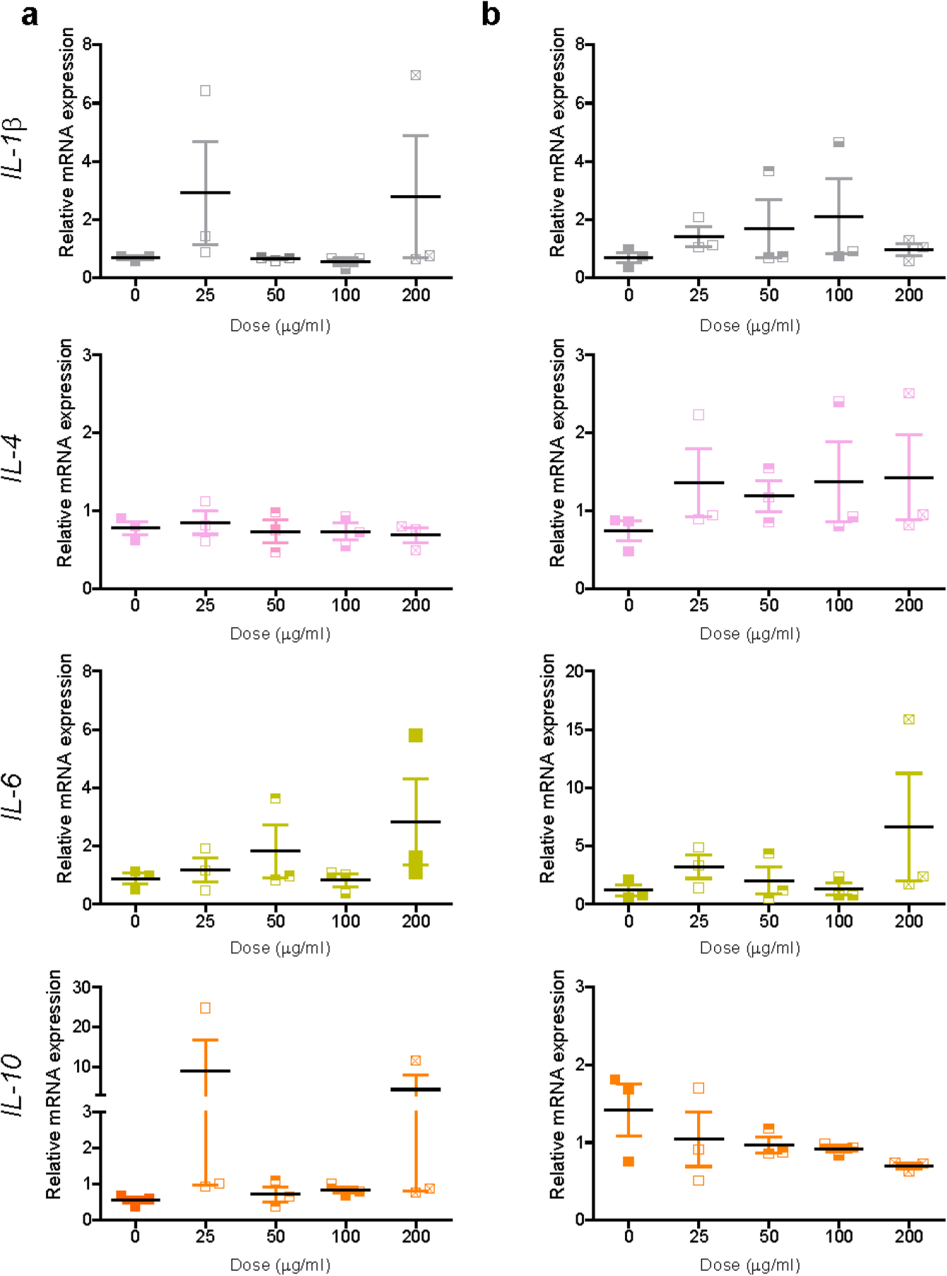
Expression of inflammation markers after TiO_2_ nanoparticle exposure. Liver slices were exposed for 72 h to TiO_2_ in WME + 5% FBS without (**a**) and with (**b**) daily refreshment of the dispersion. The relative gene expression was determined by qRT-PCR and calculated using *β-actin* as housekeeping gene. The data are presented as the fold induction over the expression levels in slices not exposed to nanoparticles, calculated as described in the “Materials and methods” section. The results are the mean and standard error of the mean of three independent experiments. Kruskal–Wallis statistic followed by Dunnett’s multiple comparisons test were performed on the Δ*Ct* values

**Fig. 9:**
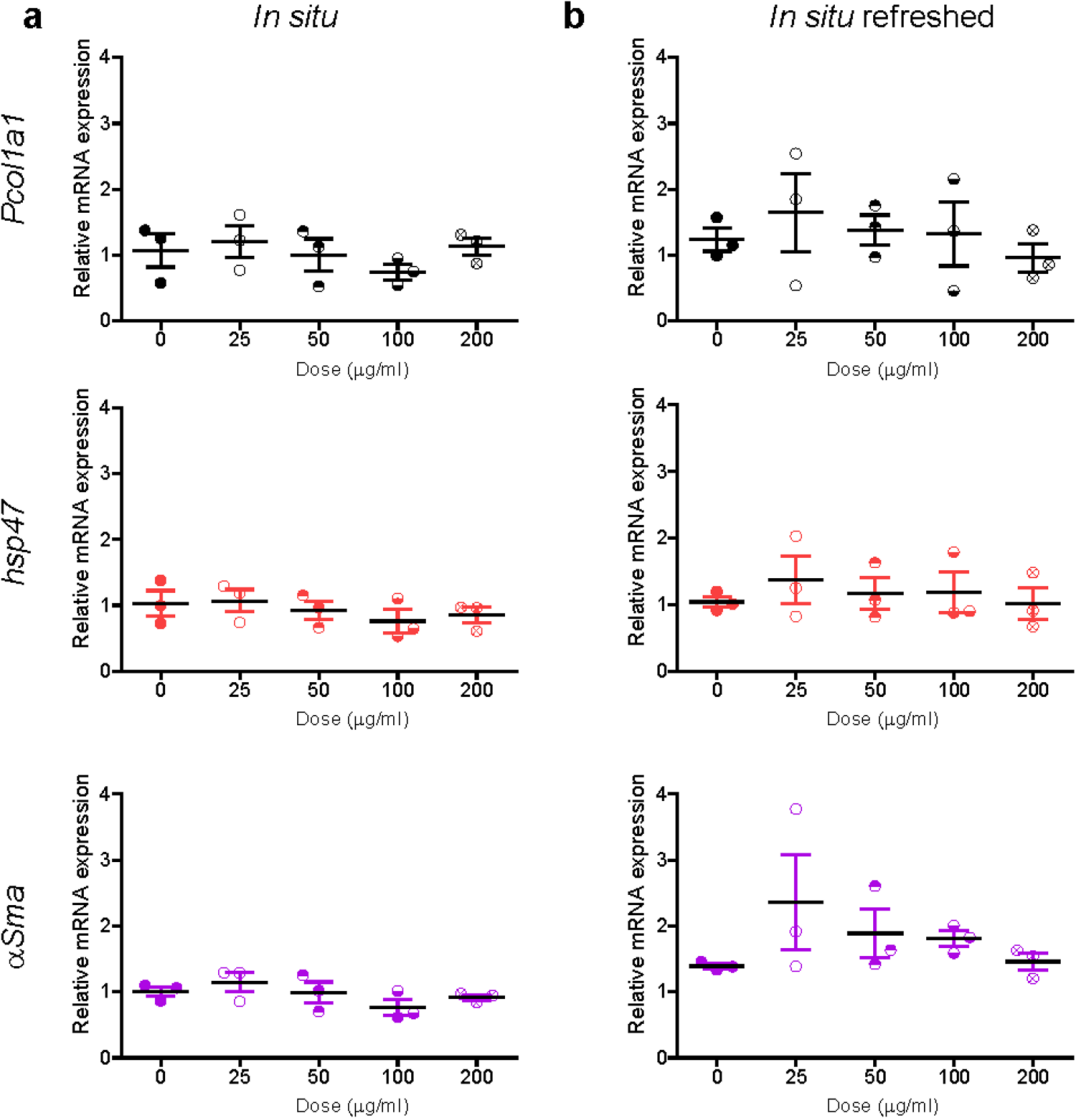
Expression of fibrosis markers after TiO_2_ nanoparticle exposure. Liver slices were exposed for 72 h to TiO_2_ in WME + 5% FBS without (**a**) and with (**b**) daily refreshment of the dispersion. The relative gene expression was determined by RT-qPCR and calculated using *β-actin* as housekeeping gene. The data are presented as the fold induction over the expression levels in slices not exposed to nanoparticles, calculated as described in the “Materials and methods” section. The results are mean o and standard error of the mean of three independent experiments. Kruskal–Wallis statistic followed by Dunnett’s multiple comparisons test were performed on Δ*Ct* values

Similar to SiO_2_ nanoparticles, titania (TiO_2_) nanoparticles were selected as another well-known model nanomaterial, broadly studied in nanosafety in relation to inflammation and fibrosis (Hong and Zhang 2016; Suker and Jasim 2018; Valentini et al. 2019). Prior to mRNA expression studies, TiO_2_ nanoparticles were characterized by DLS (Supplementary Figure S11). The DLS results showed multiple peaks, indicative of aggregation. The same was observed when nanoparticles were simply resuspended in PBS or in Milli-Q water (data not shown). Given the strong agglomeration, isolation of corona-TiO_2_ was not attempted, however to test for potential induction of fibrosis and inflammation, liver slices were exposed to the TiO_2_ nanoparticles in medium supplemented with 5% FBS with and without daily refreshing of the dispersion. No decrease of viability was observed after exposure to increasing doses of TiO_2_ nanoparticles in any of the tested conditions (Supplementary Figure S12). Similarly, no significant mRNA alterations were observed for both inflammation and fibrosis markers (Figs. 8, 9 and Supplementary Figure S13). Only a dose-dependent decrease was observed for *IL*-*10,* yet not significant (probably also because of the small number of replicates). Also in this case, collagen I and Sirius-red stainings were performed, but no evident alterations were observed (Supplementary Figure S14).

Similar to SiO_2_ nanoparticles, previous in vivo studies which reported induction of fibrosis upon exposure to TiO_2_ nanoparticles were carried out for up to a few months (Chen et al. 2009; Hong and Zhang 2016; Suker and Jasim 2018). Thus, it is likely that much longer timescales are required to detect similar responses.

## Discussion

A central discussion in the toxicity field has always been the relevance of the models that can be used for long term studies (Pearson 1986; DelRaso 1993; Holmes et al. 2010; Krewski et al. 2010; Soldatow et al. 2013; Mahony et al. 2018). Naturally, this applies also to the nanosafety field (Krug 2014; Burden et al. 2017; Drasler et al. 2017; Accomasso et al. 2018; Guggenheim et al. 2018; Singh et al. 2019). There is an urgent need to optimize and validate good models that can be used to translate acute nanoparticle responses, as generally investigated in vitro*,* and chronic effects, usually studied in vivo. Several advanced in vitro models have been explored for this purpose, but it appears difficult to identify the ideal model that could allow resembling and connecting both in vitro and in vivo results. The cellular complexity and cell architecture of complex in vivo environments remains challenging to be reproduced in vitro, while the limited timeframe that can be explored using common in vitro models represents another obstacle, and is typically even more limited for advanced in vitro systems, as also the tissue slices used for this study (here cultured for up to 72 h). Furthermore, when testing nanomaterials other important factors need to be considered to extract meaningful information, such as the inclusion of corona and additional effects due to ageing of dispersions in biological conditions (Kuchibhatla et al. 2012; Lesniak et al. 2012; Monopoli et al. 2012; Gubicza et al. 2013; Jasbi and Dorranian 2016; Lu et al. 2017). These factors, altogether, make studies of chronic nanoparticle impact in advanced models highly challenging.

Within this context, tissue liver slices represent a well-established 3D model in which the natural composition and organization of cells are maintained intact ex vivo (Olinga and Schuppan 2013). Because of this, we decided to attempt a long-term nanoparticle exposure in tissue slices to study potential impact in a more complex model. Even though this model can be used for only up to 72 h, given the natural activation of fibrosis markers observed in tissue slices during culture, we were interested to determine nanoparticle interference with the early onset of fibrosis. A panel of different nanomaterials well studied in nanosafety was selected, and inflammation and fibrosis markers were monitored.

In summary, our results showed that liver tissue slices were able to respond to known toxic and pro-inflammatory nanoparticles ex vivo, suggesting they can be used as an appropriate model to test potential inflammatory responses induced by nanoparticles. Moreover, we confirmed once more the importance of including biological fluids such as serum to allow corona formation and avoid artifacts due to direct interactions with bare particles, usually associated with toxicity (Ge et al. 2011; Lesniak et al. 2012; Duan et al. 2015). Liver tissue will always be in contact with nanoparticles modified by protein adsorption from the surrounding environment (likely serum from the systemic circulation) and a serum-free condition will never really occur in the liver in vivo. Furthermore, we demonstrated that ageing of nanoparticle dispersions in biological conditions is another factor that needs to be taken into account and explicitly tested, especially when attempting to study long-term effects of nanomaterials. Nanoparticle dispersion ageing effects were already visible over 72 h exposure, as in our case.

Nevertheless, no alteration in fibrosis markers was observed for all nanoparticles and exposure conditions tested, even with SiO_2_ and TiO_2_ nanoparticles for which in vivo studies reported the capacity to induce fibrosis in chronic exposure (Chen et al. 2009; Hong and Zhang 2016; Yu et al. 2017; Suker and Jasim 2018). Liver slices are known to spontaneously develop the early onset of fibrosis, but it is also known that if pro-fibrotic factors are added to medium, the condition is exacerbated (Westra et al. 2014b). Thus, the model is known to respond well to fibrotic stimuli. However, given the much longer timeframe for which fibrosis induced by nanoparticles was reported (Chen et al. 2009; Hong and Zhang 2016; Yu et al. 2017; Suker and Jasim 2018), it is most likely that longer exposure times should be tested to be able to observe similar effects. The maintenance of mouse liver slices has currently been optimized up to 72 h, but—thanks to further optimization of the growth conditions—it has been shown that rat and human liver slices can now be maintained in culture for up to 5 days (Starokozhko et al. 2015, 2017). It would be interesting to expand this investigation using rat and human liver slices to be able to test longer exposure times, as well as to compare species-specific differences. Similarly, coronas formed at higher serum content more closely resembling plasma protein concentrations could be tested, as well as other nanoparticles such as copper oxide, zinc oxide, silver and ceria nanoparticles, which may have different modes of action and may show impact on the tissue already at 72 h.

In conclusion, in the present study we show that precision-cut tissue liver slices are a model with great potential to study nanoparticle impact in a better in vivo-like environment, opening up new opportunities to discriminate various effects involved in nanoparticle exposure in real tissue. While we show that tissue slices can be used to detect inflammatory responses, fibrotic responses induced by nanoparticles have been reported only after much longer exposure times than what is currently possible with this model. Developing appropriate models for long-term studies while reproducing the complexity of real tissue in vitro remains an important challenge and urgent need in the field, to be able to answer on potential long-term effects on nanoparticles.

## Supplementary Information

Below is the link to the electronic supplementary material.

Supplementary file1 (DOCX 14661 KB)
