# Supplementary material for: Nanoparticle-induced inflammation and fibrosis in ex vivo murine precision-cut liver slices and effects of nanoparticle exposure conditions
Source: Arch Toxicol. 2021 Feb 8;95(4):1267–85. doi: 10.1007/s00204-021-02992-7 (PMC8032640; doi:10.1007/s00204-021-02992-7)
Supplement: Supplementary file 1 — Supplementary file1 (DOCX 14661 KB) [file 204_2021_2992_MOESM1_ESM.docx]

**Supplementary Information**

**Nanoparticle-induced inflammation and fibrosis in *ex vivo* murine precision-cut liver slices and effects of nanoparticle exposure conditions**

*Roberta Bartucci, Alex Z. van der Meer, Ykelien L. Boersma, Peter Olinga, Anna Salvati*


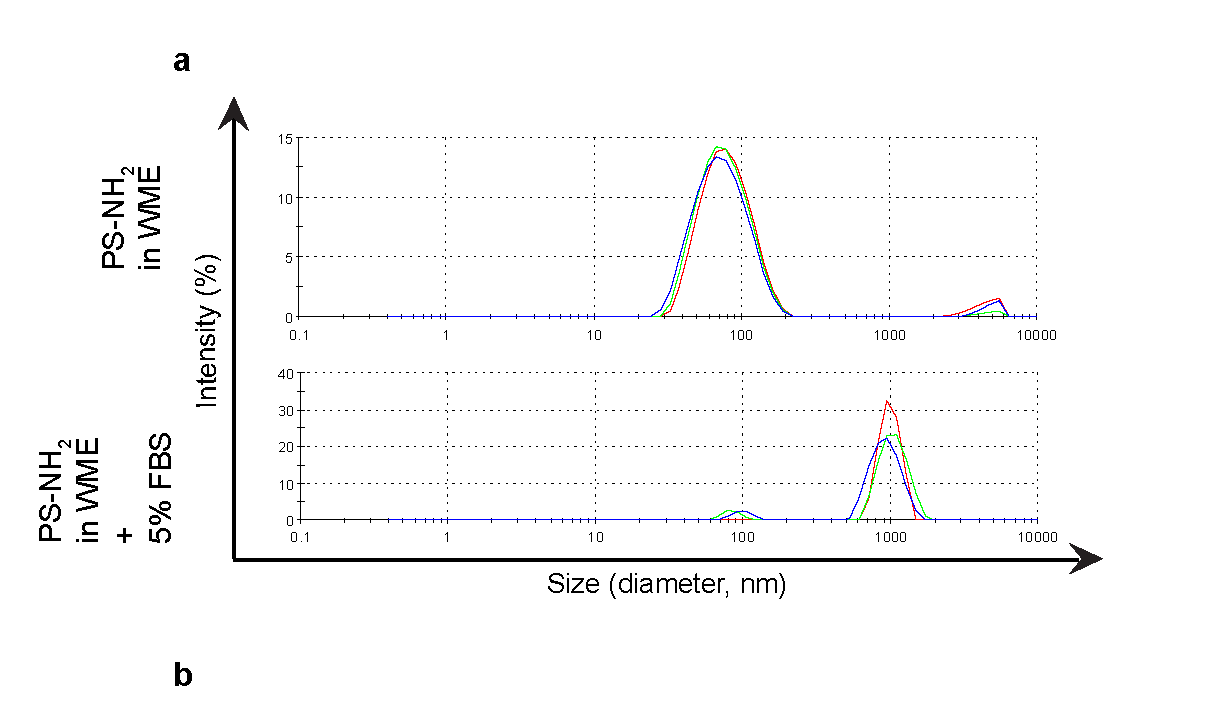


|  | **Medium** | **Diameter**  **(z-average, nm)^a^** | **PDI^a^** | **Diameter (nm)^b^** |
| --- | --- | --- | --- | --- |
| **PS-NH_2_** | WME | - | - | 80 |
| **PS-NH_2_** | WME + 5% FBS | * | * | * |

* Agglomeration

**^a^**Average hydrodynamic diameter (z-average) and polydispersity index (PDI) of nanoparticle dispersions in relevant media obtained from cumulant fitting of Dynamic Light Scattering data.

**^b^**Average hydrodynamic diameter determined from CONTIN size distribution.

**Supplementary Figure S1.** PS-NH_2_ nanoparticle dispersion characterization by dynamic light scattering (DLS). (a) Size distributions of nanoparticle dispersions obtained from CONTIN analysis of DLS data and (b) corresponding diameters, together with results obtained by cumulant fitting. The nanoparticle dispersions (100 µg/ml) in medium supplemented with 5% FBS (WME + 5% FBS) and in serum-free medium (WME) were characterized by DLS, immediately after dispersion. The results are the average of three separate measurements from a representative experiment. The PS-NH_2_ nanoparticle dispersion in WME showed a main peak around 80 nm, but also some agglomerates around 10.000 nm. PS-NH_2_ nanoparticles dispersed in WME + 5% FBS showed agglomeration, possibly because of the low content of FBS.

**Supplementary Figure S2**. Viability of rat liver slices exposed to PS-NH_2_ nanoparticles in daily-refreshed serum-free medium. Liver slices were maintained in culture up to 72 h in serum-free medium (WME) refreshed daily and exposed to PS-NH_2_ nanoparticles in serum-free WME (10 and 25 µg/ml). The viability is expressed as the ATP content (pmol) normalized by total protein amount (µg). In each independent experiment, three replicate slices treated in the same way were used and their average calculated. The results of three independent animal experiments are shown together with their mean (indicated with a line) and standard error of the mean. Exposure to these nanoparticles in serum-free medium led to strong loss of viability.


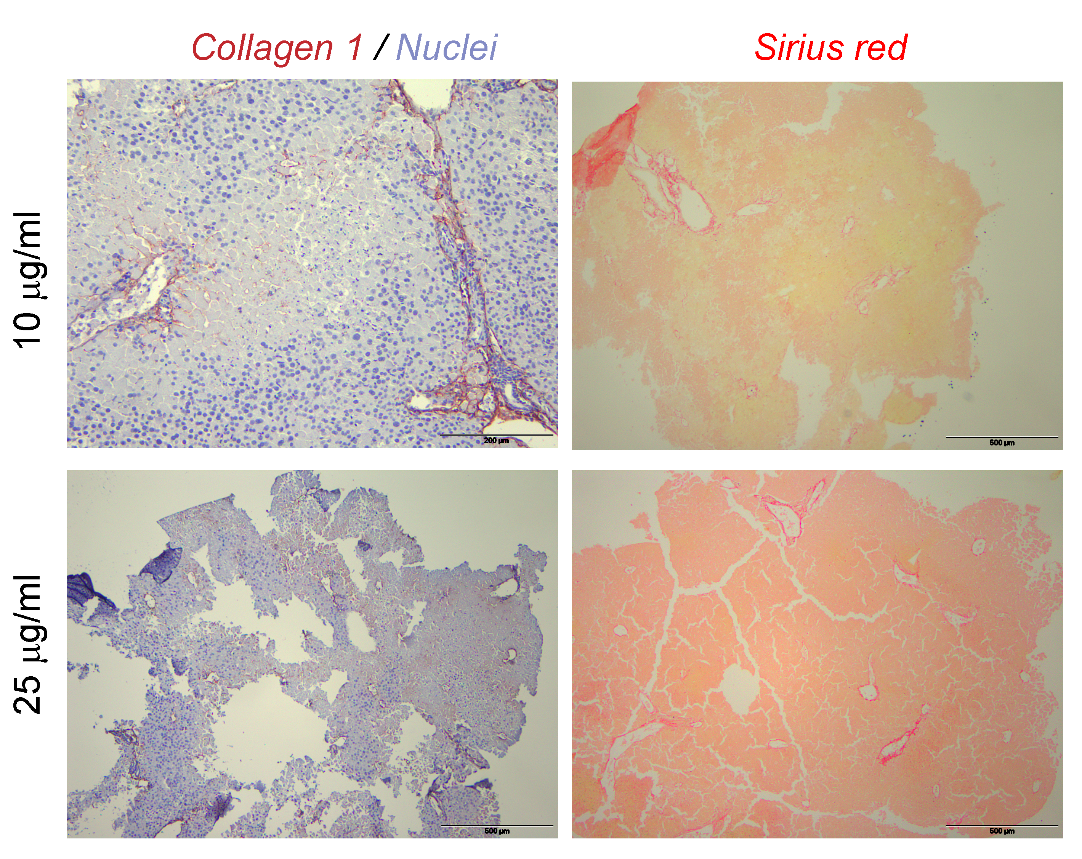


**Supplementary Figure S3***.* Collagen 1 immunostaining and sirius red staining of representative liver slices exposed to PS-NH_2_ nanoparticles. Collagen 1 immunostaining (Red= collagen 1; Violet= Nuclei; Scale bar= 200 and 500 µm, in the sections treated with 10 and 25 µg/ml, respectively) and Sirius red staining (Red= collagen fibers. Scale bar= 500 µm) of paraffin sections of liver slices exposed for 72 h to PS-NH_2_ nanoparticles (10 and 25 µg/ml*) in situ* in medium supplemented with 5% FBS (WME + 5% FBS). No evident changes in collagen staining could be observed.


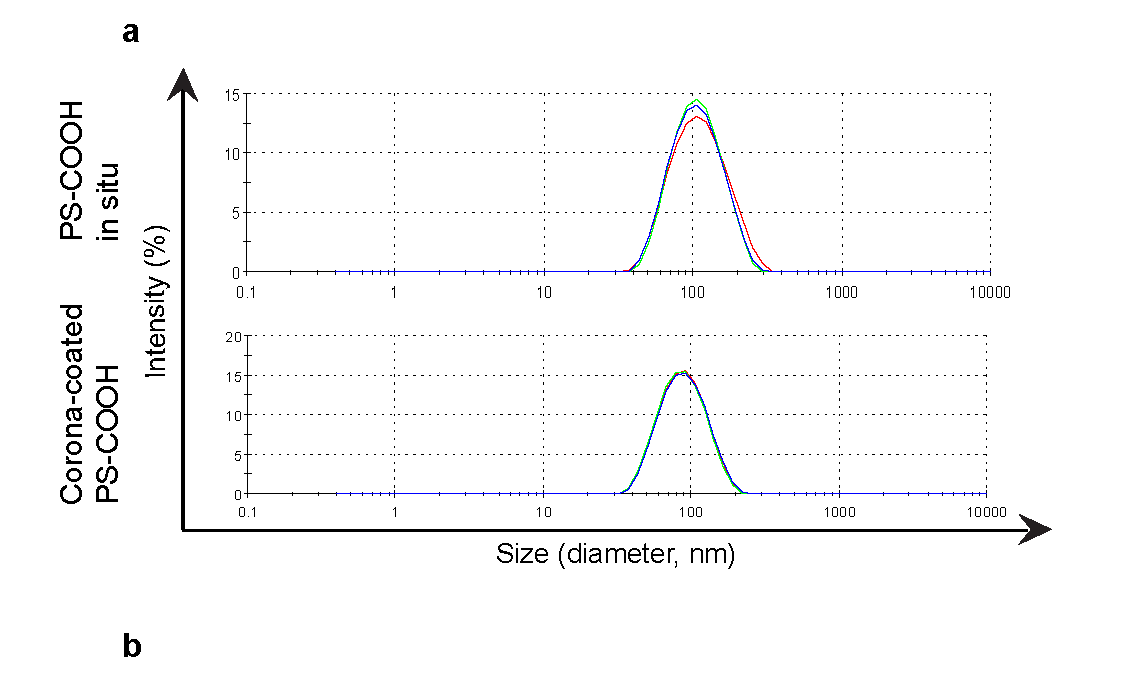

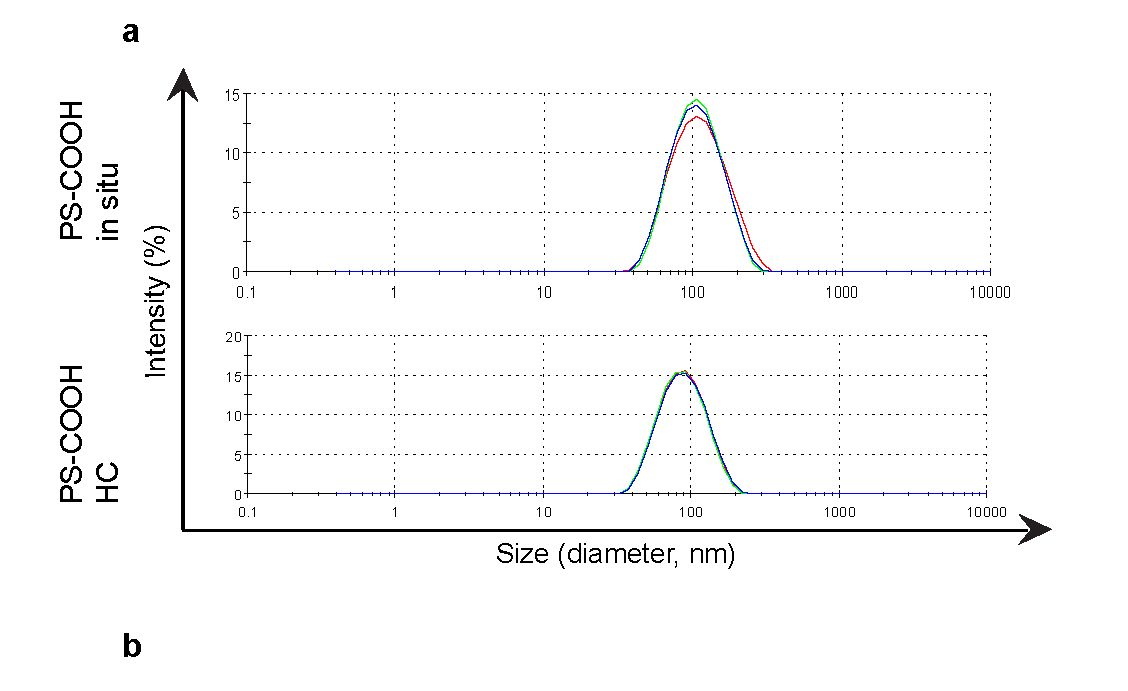


|  | **Medium** | **Diameter**  **(z-average, nm)^a^** | **PDI^a^** |
| --- | --- | --- | --- |
| **PS-COOH** | WME + 5% FBS | 95 | 0.01 |
| **Corona-coated PS-COOH** | WME | 82 | 0.01 |

* Agglomeration

**^a^**Average hydrodynamic diameter (z-average) and polydispersity index (PDI) of nanoparticle dispersions in relevant media obtained from cumulant fitting of Dynamic Light Scattering data.

**Supplementary Figure S4** PS-COOH nanoparticle dispersion characterization by dynamic light scattering (DLS). (a) Size distributions of nanoparticle dispersions obtained from CONTIN analysis of DLS data and (b) corresponding diameters, together with results obtained by cumulant fitting. The nanoparticle dispersions (100 µg/ml) in medium supplemented with 5% FBS (WME + 5% FBS) and corona-coated PS-COOH complexes were characterized by DLS, immediately after dispersion. The results are the average of three separate measurements from a representative experiment. The PS-COOH nanoparticle dispersion in WME + 5% FBS showed a peak around 95 nm and corona-coated PS-COOH nanoparticles showed a peak around 82 nm. In both cases a homogenous dispersion was observed.





**Supplementary Figure S5**. Viability of rat liver slices after 72 h of exposure to PS-COOH nanoparticles. Liver slices were maintained in culture for 72 h in serum-free medium (WME) or medium supplemented with 5% FBS (WME + 5% FBS) and exposed to PS-COOH nanoparticles (25 and 100 µg/ml) *in situ* (**a**) or to corona-coated PS-COOH nanoparticles (25 and 100 µg/ml) (**b**). The viability is expressed as the ATP content (pmol) normalized by total protein amount (µg). In each independent experiment, three replicate slices treated in the same way were used and their average calculated. The results obtained in three independent animal experiments are shown together with their mean (indicated with a line) and standard error of the mean. No impact on cell viability was observed in all conditions tested.

**
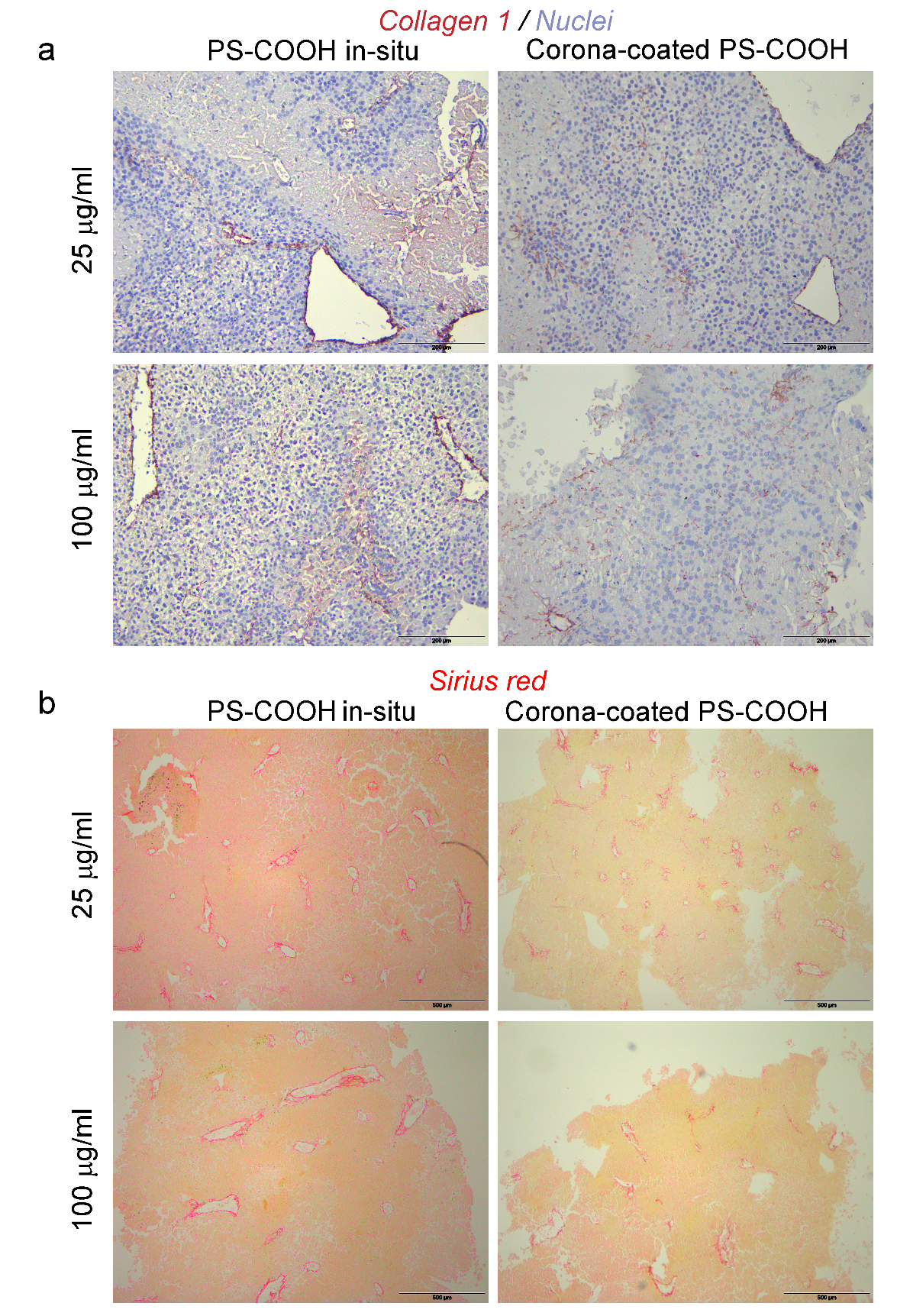
**

**Supplementary Figure S6*.*** Collagen 1 immunostaining and sirius red staining of representative liver slices exposed to PS-COOH nanoparticles. (a) Collagen 1 immunostaining and (b) sirius red staining of paraffin sections of liver slices exposed for 72 h to PS-COOH nanoparticles (25 and 100 µg/ml) *in situ* and to corona-coated PS-COOH nanoparticles (25 and 100 µg/ml). Red= collagen 1; Violet= Nuclei. Scale bar= 200 µm. (b) Red= collagen fibers. Scale bar= 500 µm. No evident changes in collagen staining could be observed.


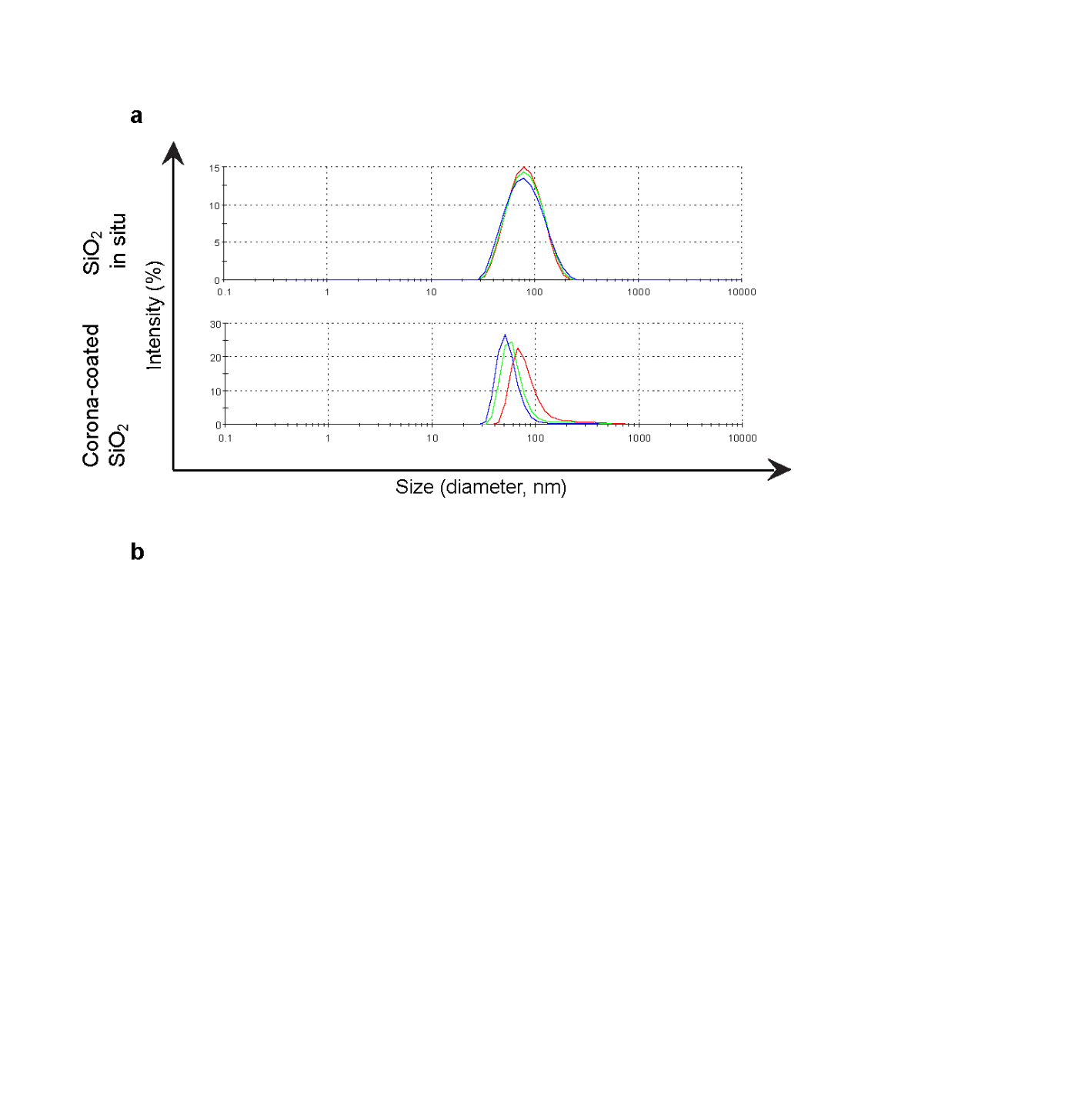


|  | **Medium** | **Diameter**  **(z-average, nm)^a^** | **PDI^a^** |
| --- | --- | --- | --- |
| **SiO_2_ in situ** | WME + 5% FBS | 65 | 0,14 |
| **Corona-coated SiO_2_** | WME | 74 | 0,12 |

* Agglomeration

**^a^**Average hydrodynamic diameter (z-average) and polydispersity index (PDI) of nanoparticle dispersions in relevant media obtained from cumulant fitting of Dynamic Light Scattering data.

**Supplementary Figure S7.** SiO_2_ nanoparticle dispersion characterization by dynamic light scattering (DLS). (a) Size distributions of nanoparticle dispersions obtained from CONTIN analysis of DLS data and (b) corresponding diameters, together with results obtained by cumulant fitting. The nanoparticle dispersions (100 µg/ml) *in situ* and corona-coated SiO_2_ nanoparticles were characterized by DLS, immediately after dispersion. The results are the average of three separate measurements from a representative experiment. The SiO_2_ nanoparticle dispersion *in situ* showed a main peak around 65 nm and corona-coated SiO_2_ nanoparticles around 74 nm. In both cases a homogenous dispersion was formed.





**Supplementary Figure S8**. Viability of rat liver slices after 72 h of exposure to SiO_2_ nanoparticles. Liver slices were maintained in culture for 72 h in serum-free medium (WME) or medium supplemented with 5% FBS (WME + 5% FBS) and exposed to SiO_2_ nanoparticles (100 µg/ml) *in situ* (**a**) or to corona-coated SiO_2_ nanoparticles (50 and 200 µg/ml) (**b**). The viability is expressed as the ATP content (pmol) normalized by total protein amount (µg). In each independent experiment, three replicate slices treated in the same way were used and their average calculated. The results obtained in three independent animal experiments are shown together with their mean (indicated with a line) and standard error of the mean. No impact on cell viability was observed in all conditions tested.

**
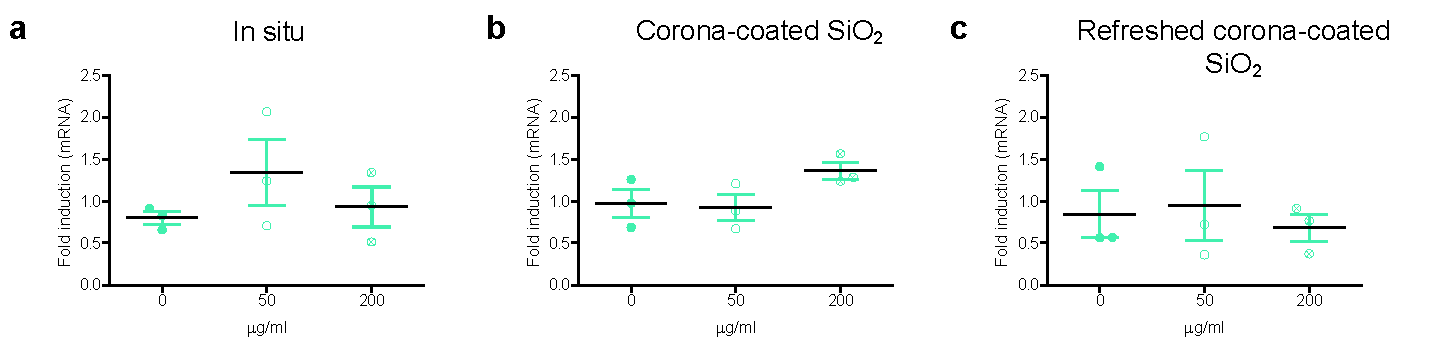
Supplementary Figure S9.**  mRNA expression of *Tgf-β* after SiO_2_ nanoparticle exposure. Liver slices were exposed for 72 h to SiO_2_ nanoparticles *in situ* (a), or to corona-coated SiO_2_  nanoparticles non-daily refreshed (b) and with daily refreshment of the dispersion (**c**). The relative mRNA expression was determined by qRT-PCR and calculated using *β-actin* as housekeeping gene as described in the Methods. The mean of and standard error of the mean of the results obtained in three independent experiments are shown. Kruskal-Wallis statistic followed by Dunnett’s multiple comparisons test were performed on ΔCt values. No evident alteration of *Tgf-β* expression was observed in all conditions tested.

**
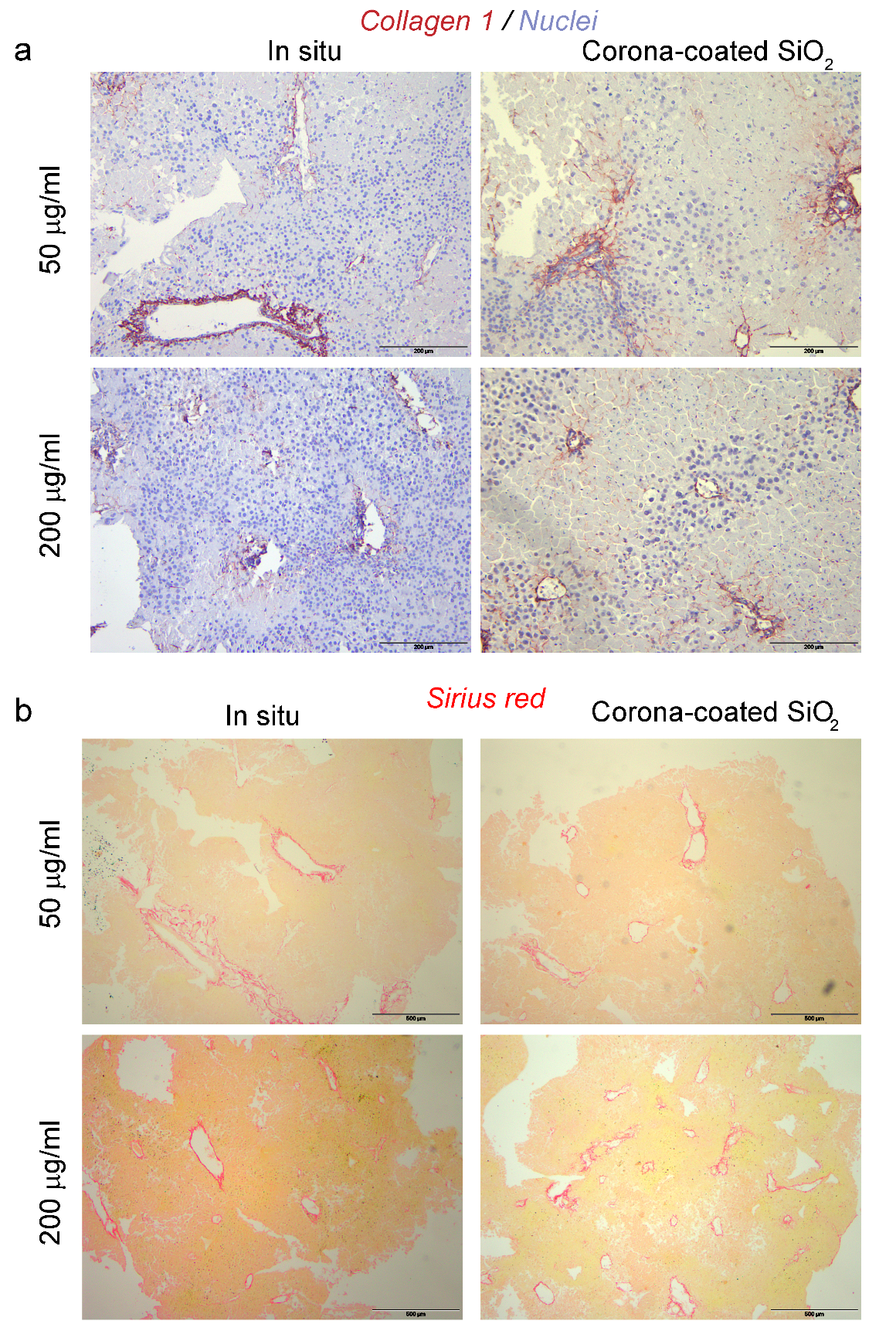
Supplementary Figure S10***.* Collagen 1 immunostaining and sirius red staining of representative liver slices exposed to SiO_2_ nanoparticles. (a) Collagen 1 immunostaining and (b) sirius red staining of paraffin sections of liver slices exposed for 72 h to SiO_2_ nanoparticles (50 and 200 µg/ml) *in situ* and to corona-coated SiO_2_ (50 and 200 µg/ml). Red= collagen 1; Violet= Nuclei. Scale bar= 200 µm. (b) Red= collagen fibers. Scale bar= 500 µm. No evident changes in collagen staining could be observed.


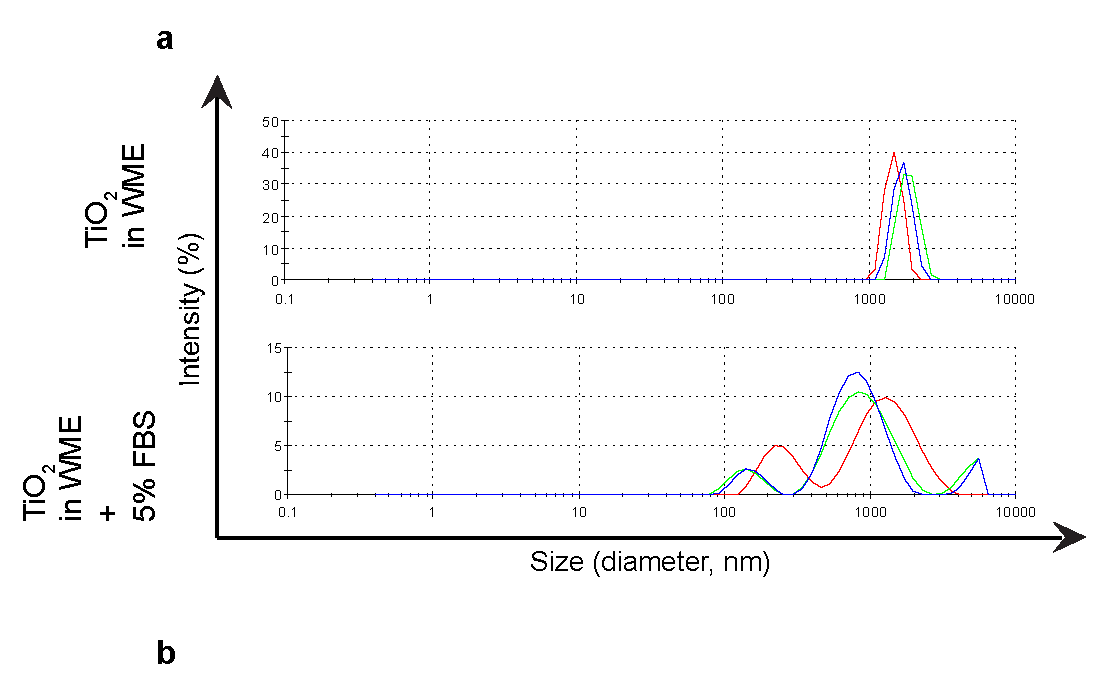


**Supplementary Figure S11.** TiO_2_ nanoparticle dispersion characterization by dynamic light scattering. (a) Size distributions of nanoparticle dispersions obtained from CONTIN analysis of DLS data. The nanoparticle dispersions (500 µg/ml) in medium supplemented with 5% FBS (WME + 5% FBS) and in serum-free medium (WME) were characterized by DLS, immediately after dispersion. The results are the average of three separate measurements from a representative experiment. The TiO_2_ nanoparticle dispersion in WME showed a peak at sizes larger than 1000 nm, sign of agglomeration (the nominal particle size was 8 nm). Aggregation was also observed for TiO_2_ nanoparticles in WME + 5% FBS.

**Supplementary Figure S12**. Viability of rat liver slices after 72 h of exposure to increasing doses of TiO_2_ nanoparticles. Liver slices were maintained in culture up to 72 h in serum-free medium (WME) or medium supplemented with 5% FBS (WME + 5% FBS) and exposed for 72 h to TiO_2_ nanoparticles (25, 50, 100 and 200 µg/ml) in WME + 5% FBS. The viability is expressed as the ATP content (pmol) normalized by total protein amount (µg). In each independent experiment, three replicate slices treated in the same way were used and their average calculated. The results of three independent animal experiments are shown together with their mean (indicated with a line) and standard error of the mean. .


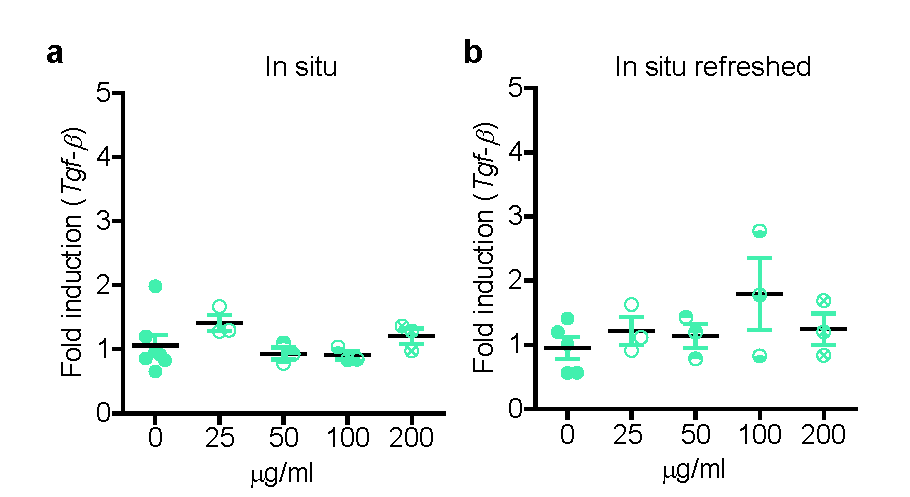


**Supplementary Figure S13.**  mRNA expression of *Tgf-β* after TiO_2_ nanoparticle exposure. Liver slices were exposed for 72 h to TiO_2_ nanoparticles in WME + 5% FBS *in situ* without (a) and with (b) daily refreshment of the dispersion. The relative mRNA expression was determined by RT-qPCR and calculated using *β-actin* as housekeeping gene as described in the Methods. The mean and standard error of the mean of the results obtained in three independent experiments are shown. Kruskal-Wallis statistic followed by Dunnett’s multiple comparisons test were performed on ΔCt values. No evident alteration of *Tgf-β* expression was observed in all conditions tested.


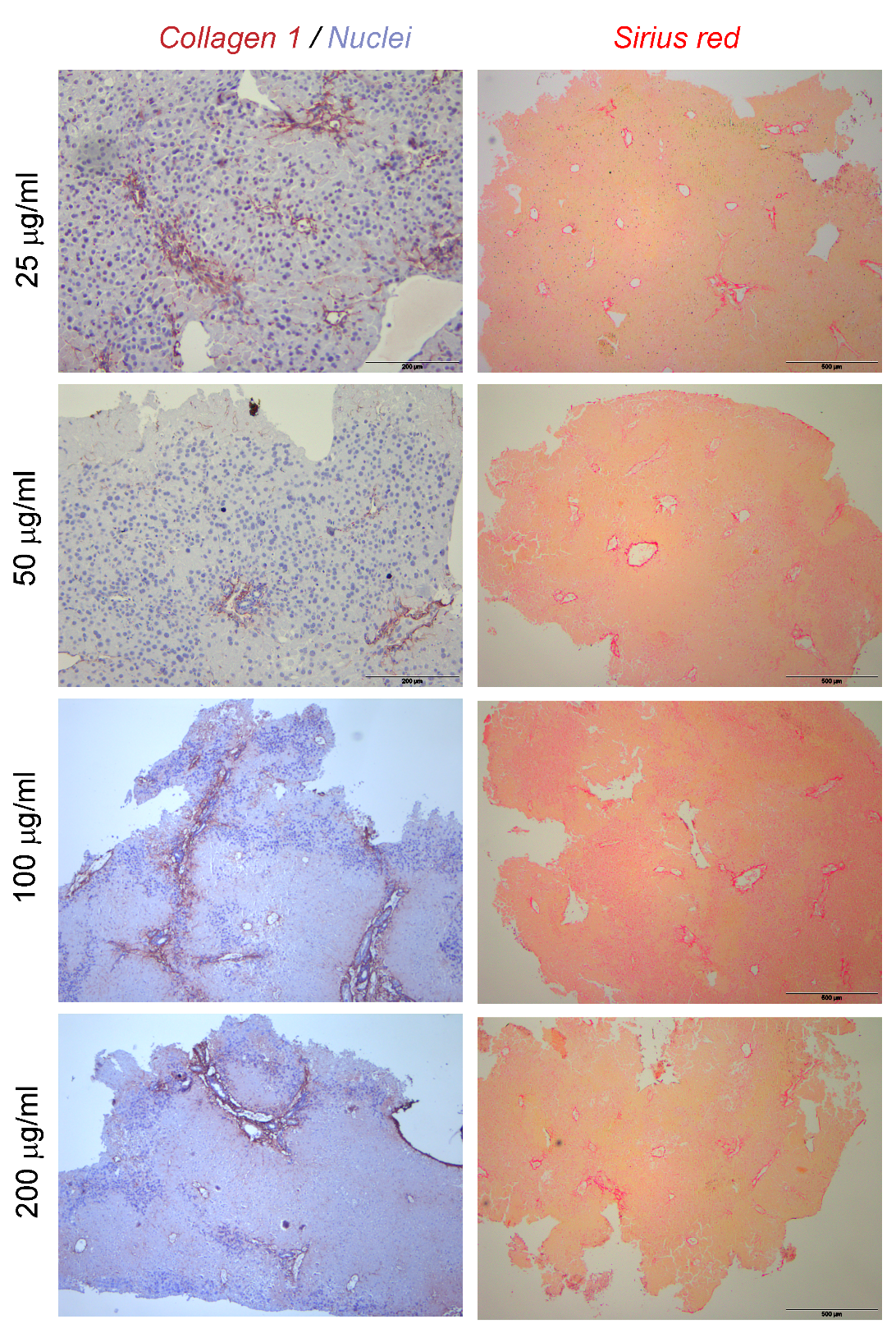


**Supplementary Figure S14***.* Collagen 1 immunostaining and sirius red staining of representative liver slices exposed to increasing doses of TiO_2_ nanoparticles. Collagen 1 immunostaining and sirius red staining of paraffin sections of liver slices exposed for 72 h to TiO_2_ nanoparticles (25, 50, 100 and 200 µg/ml) in medium supplemented with 5% FBS (WME + 5% FBS) *in situ*. Left side: Red= collagen 1; Violet= Nuclei. Scale bar= 200 µm. Right side: red= collagen fibers; scale bar= 500 µm. No evident changes in collagen staining could be observed.
